# Adaptation of *Drosophila* to a novel laboratory environment reveals temporally heterogeneous trajectories of selected alleles

**DOI:** 10.1111/j.1365-294X.2012.05673.x

**Published:** 2012-10

**Authors:** Pablo Orozco-terWengel, Martin Kapun, Viola Nolte, Robert Kofler, Thomas Flatt, Christian Schlötterer

**Affiliations:** Institut für PopulationsgenetikVetmeduni Vienna, Veterinärplatz 1, A-1210 Vienna, Austria

**Keywords:** adaptation, laboratory evolution, selective trajectories

## Abstract

The genomic basis of adaptation to novel environments is a fundamental problem in evolutionary biology that has gained additional importance in the light of the recent global change discussion. Here, we combined laboratory natural selection (experimental evolution) in *Drosophila melanogaster* with genome-wide next generation sequencing of DNA pools (Pool-Seq) to identify alleles that are favourable in a novel laboratory environment and traced their trajectories during the adaptive process. Already after 15 generations, we identified a pronounced genomic response to selection, with almost 5000 single nucleotide polymorphisms (SNP; genome-wide false discovery rates < 0.005%) deviating from neutral expectation. Importantly, the evolutionary trajectories of the selected alleles were heterogeneous, with the alleles falling into two distinct classes: (i) alleles that continuously rise in frequency; and (ii) alleles that at first increase rapidly but whose frequencies then reach a plateau. Our data thus suggest that the genomic response to selection can involve a large number of selected SNPs that show unexpectedly complex evolutionary trajectories, possibly due to nonadditive effects.

## Introduction

One of the central goals in evolutionary biology is to understand adaptation. The different genetic approaches used to study adaptation can be broadly grouped into three categories: (i) QTL mapping; (ii) population genetics; and (iii) experimental evolution. For many years, quantitative genetics has been the workhorse of evolutionary biologists who study the genetic architecture of traits thought to be associated with adaptation. Such QTL studies have shed light on the number of genes contributing to a trait, the distribution of effect sizes, dominance and the identification of the causative mutation(s) (Erickson *et al.* 2004; Mackay *et al.* 2009). More recently, genome-wide association studies have become popular (Hirschhorn & Daly 2005; Atwell *et al.* 2010). These no longer require experimental crosses but instead take advantage of historic recombination events that break up linkage disequilibrium (LD). While QTL mapping and association mapping have their specific strengths and weaknesses, the major limitation of both methods is that they require a priori information about the adaptive nature of a given trait. The population genetic approach, in contrast, does not require any a priori information about the selected trait. Instead, targets of selection are identified by contrasting observed data with expectations based on the population genetic theory (Schlötterer 2003; Nielsen 2005). While this approach is conceptually appealing, it has become increasingly clear that complex demographic histories that involve migration and population bottlenecks can generate a signature in the genome that cannot be distinguished from selection (Thornton *et al.* 2007; Parsch *et al.* 2009).

A completely different approach towards understanding adaptation is pursued by experimental evolution (Garland & Rose 2009). This method not only controls the environment and population history, but also allows for replication. Experimental evolution has been successfully used in microorganisms, such as *Escherichia coli* (Elena & Lenski 2003) and yeast (Zeyl 2006; Parts *et al.* 2011), and also in multicellular organisms, including *Drosophila* (Hoffmann *et al.* 2003; Turner *et al.* 2011; Zhou *et al.* 2011). Given the very large population sizes of microorganisms in laboratory experiments and their short generation times, the standard procedure has been to start from a single clone and study the effects of new mutations that have accumulated during the experiment. For example, the trajectory of adaptation over 40 000 generations was recently analysed on the genomic scale in *E. coli*, with the surprising result that the rate of novel beneficial mutations remained constant, whereas adaptation decelerated markedly (Barrick *et al.* 2009). In contrast, allele frequency changes (AFCs), rather than new mutations, fuel experimental evolution in multicellular organisms (Burke *et al.* 2010). In these organisms, the experimenter subjects polymorphic experimental populations to either truncating (artificial) selection or to laboratory natural selection. In the latter, populations are exposed to a defined environment for multiple generations and, as in nature, fitness differences among individuals result in adaptation.

Most experimental evolution studies in *Drosophila* have focused on the response of various phenotypic traits to selection. Thus, despite a large number of studies, very little is known about the underlying genetic trajectories. Studies that have pioneered the analysis of the genetic signature in experimental *Drosophila* populations have used allozymes and later microsatellites (Rand *et al.* 2010) or single nucleotide polymorphisms (SNPs; Teotónio *et al.* 2009). Although these markers only covered a tiny fraction of the genome, at least one marker displaying a pattern of non-neutral evolution was detected in each study. The consistent identification of selection with a moderate number of markers strongly suggests that a large fraction of the genome responds to selection.

With the advent of second-generation sequencing technology, it has become possible to sequence multiple individuals on a whole-genome scale. In particular, sequencing of DNA from pooled individuals (Pool-Seq) provides an excellent tool to determine allele frequencies on a genomic scale (Futschik & Schlötterer 2010; Kolaczkowski *et al.* 2011). Through the comparison of allele frequencies between two populations, selected SNPs can be identified. To date, only few studies have reported the sequencing of differentially selected populations. Apart from studies in chicken (Rubin *et al.* 2010) and *Arabidopsis* (Turner *et al.* 2010), three studies focussed on *Drosophila melanogaster*. One of them compared populations from a long-term selection experiment and concluded that even during 600 generations of laboratory evolution novel mutations did not contribute to adaptation of the populations. The adaptive divergence of the populations was entirely based on frequency changes from standing variation (Burke *et al.* 2010). The other two studies analysed the outcome of truncating selection for hypoxia tolerance (Zhou *et al.* 2011) and body size (Turner *et al.* 2011). Both studies convincingly identified a large number of selected SNPs. For hypoxia tolerance, some of the identified candidates were even functionally validated, demonstrating the power of laboratory selection for identifying adaptive candidate genes. However, with the exception of a single study in yeast (Parts *et al.* 2011), the published reports using second-generation sequencing have not yet fully exploited the power of experimental evolution because they only compared evolved populations: neither the ancestral population nor multiple time points were analysed.

Here, we analyse multiple time points in experimental evolution populations of *D. melanogaster* adapting to a novel environment and identify unexpectedly complex evolutionary trajectories of selectively favoured alleles: one class raises quickly in frequency, but reaches a plateau, whereas the other class shows a continuous change in allele frequency.

## Material and methods

### Drosophila melanogaster population sample

We generated isofemale lines from a fresh collection of *Drosophila melanogaster* from northern Portugal (Povoa de Varzim) in 2008. The isofemale lines were kept in the laboratory for five generations before the start of the experiment to acclimate flies, confirm their species status and identify lines infected with parasites. We generated three independent replicate base (B) populations by sampling for each replicate five nonvirgin females from a total of 113 isofemale lines (=565 females per replicate in total). These females were divided among five 8 oz bottles containing 70 mL of standard *Drosophila* medium.

### Culture conditions

Approximately 3 days after eclosion, 1000 adults were randomly selected (assuming a 50:50 sex ratio) to generate a new generation. After 48 h of egg-laying, adults were transferred to five fresh bottles to lay eggs for another 48 h, after which flies were discarded or used for DNA extraction. The first set of bottles was used as a backup in case that the second set did not yield a sufficient number of flies. The flies were cultured in a fluctuating temperature and light regime to mimic natural conditions: 12 h at 18 °C (dark) and 12 h at 28 °C (light).

### Inversion analysis

We determined the frequency change of *In(3R)P* by using a PCR-based assay, based on a modification of a previously described method (Anderson *et al.* 2005). We used the following primers: ACTAGCGTTGAGAATGCAAAGTCCAAC (P1), AAATGCTGCACGTAATTGTAAGTTATGAGC (P2) and ACAACTTTTGGCACGCGAATT (P6). P1 and P2 provide a positive control for the PCR and amplify a PCR product of 306 bp. P2 in combination with P6 amplifies a band of 663 bp if the inversion is present. The PCR conditions for this reaction were as follows: 1 min at 94 °C, followed by 30 cycles of 30 s at 94 °C, 30 s at 54 °C and 90 s at 72 °C, with a final step of 5 min at 72 °C. Fifteen microlitre PCR reactions were carried out with 50 ng genomic DNA, 1 μm primer, 2.5 mm MgCl_2_, 200 μm dNTPs and 1 U Taq polymerase. The PCR products were separated on 1% agarose gels and stained with ethidium bromide.

We assayed 100 individuals from each of the following samples: the base population, one replicate from generation 18, and two replicates from generation 38. As all frozen flies from the base population were homogenized for sequencing, we re-constituted the base population from the isofemale lines that were used to initiate the experimental evolution experiment.

### Sequencing

For genomic DNA preparation, a pool of 500 females was homogenized with an Ultraturrax T10 (IKA-Werke, Staufen, Germany), and DNA was extracted from the homogenate using the Qiagen DNeasy Blood and Tissue Kit (Qiagen, Hilden, Germany). We sheared the genomic DNA using a Covaris S2 device (Covaris, Inc. Woburn, MA, USA) and prepared paired-end libraries using the Paired-End DNA Sample Preparation Kit (Illumina, San Diego, CA, USA) following the manufacturer’s instructions. Paired-end reads were sequenced on a gaiix sequencer and fastq files were produced with Illumina pipeline version 1.4.

We sequenced three replicates at three time points during the experiment: (i) three replicates of the base population at the beginning of the experiment (B); (ii) two replicates at generation 15 and the third replicate at generation 23 at the middle of the experiment (M); and (iii) three replicates at the end of the experiment (E, generation 37). In addition, we also sequenced a single replicate for generation 27.

### Mapping of reads

As previously described (Kofler *et al.* 2011), we trimmed the reads to remove low quality bases, mapped them with bwa (version 0.5.7; Li & Durbin 2009) against the *D. melanogaster* reference genome (version 5.18) and *Wolbachia* (NC_002978.6). We used the following mapping parameters: −n 0.01 (error rate), −o 2 (gap opening), −d 12 and −e 12 (gap length), and −l 150 to disable the seed option. The alignment files were converted to the SAM format using the BWA module sampe enabling a local alignment procedure (Smith-Waterman) whenever one of the reads of the pair could not be mapped with global alignment. SAM files were filtered for reads mapped in proper pairs with a minimum mapping quality of 20 using SAMtools (Li *et al.* 2009). The filtered SAM files were converted into the pileup format. We used repeatmasker 3.2.9 (http://www.repeatmasker.org) to create a gff file to mask simple sequence repeats and transposable elements of the *D. melanogaster* genome version 5.34. Finally, indels together with five flanking nucleotides (on both sides) were masked in the alignments of each population if the indel was present in at least one population and supported by at least two reads.

### Effective population size

We used the temporal changes in allele frequencies from the comparison of the B, M and E populations to estimate the effective population size (using the method of Bollback *et al.* 2008). As the estimation procedure is CPU intensive, we sampled 1000 representative SNPs and traced their AFC throughout the entire experiment.

### SNP calling

Only SNPs were considered that met the following quality criteria: (i) occurrence in at least two replicate populations; (ii) the minor allele was covered by at least 10 reads across all six populations analysed; (iii) the maximum coverage did not exceed 500. A region of 1 Mb length on *3R* was excluded from the analysis as a low-frequency haplotype spreads during the experiment. We identified this haplotype by SNPs that were not detected in the base population, but which occurred at moderate frequency in the evolved populations. As this increase in frequency is most likely caused by a single beneficial mutation, we did not include this region in our analysis. Additionally, SNPs located in the proximity of the chorion gene cluster on *3L* were also excluded because this region is amplified up to 60- to 100-fold during oogenesis (Orr-Weaver & Spradling 1986), which results in false-positive candidates because of high coverage.

### Identification of candidate SNPs

We used the Cochran–Mantel–Haenszel (CMH) test to identify SNPs with an AFC between different time points that was consistent among replicates. The CMH test is used to test 2 × 2 × *k* contingency tables (where *k* is the number of independent replicates) for independence of marginal sums across *k* replicates. Under the null hypothesis, odds ratios for each replicate are not different from one (i.e. if the allele frequencies at two time points are the same; McDonald 2009). The statistic asymptotically follows a *χ*^2^ distribution with one degree of freedom (Agresti 2002). CMH tests were performed on a SNP-wise basis for the comparisons of generations B-M, B-E and M-E.

The CMH test only tests for significant allele frequency differences between generations. We therefore performed computer simulations using a simple Wright–Fisher model of neutral evolution to estimate the degree to which the observed AFC could be explained by drift alone. The Wright–Fisher model assumes an infinite haploid population with a constant number *N* of surviving offspring with only two alleles *A* and *a* at frequencies p(*A*) and p(*a*). At each generation, a random number *j* is drawn from a binomial distribution with parameters *N* and p(*A*), resulting in a new p(*A*)_t+1_ by dividing *j* by *N*. This process is repeated for each following generation (Otto & Day 2007).

Forward simulations were designed to match the experimental data as closely as possible. Thus, at the beginning of the simulations, we used the allele frequencies obtained from the base population to estimate p(*A*) and the effective population size computed for the real data to estimate the effective population size *N*. Our simulations were based on the same number of replicates and generations as in the real data. In addition, we accounted for heterogeneity in coverage in the Pool-Seq data and sampled for every SNP the same number of reads as in the real data sets by drawing from a binomial distribution with the simulated allele frequency. These simulated data were subjected to the same filtering procedures and to the CMH test as used for the experimental data. To correct for multiple testing, we calculated empirical false discovery rates (FDR) by defining a *P*-value threshold based on highest *P*-value of the top 0.001% from simulated SNPs. We then used this threshold as a cut-off for the experimental data to identify significant loci based on the simulations. An approximate FDR was calculated by dividing the count of the top 0.001% simulated SNPs by the number of experimental SNPs below the *P*-value threshold, that is, the ratio of false-positives because of drift to selected candidate SNPs. For the top 2000 candidates, we used the maximum *P*-value of the experimental candidate data to count the number of simulations below this threshold and calculated the FDR as described above.

### Feature analysis

We used snpeff 2.0.1 (http://snpeff.sourceforge.net/) and the *D. melanogaster* annotation version 5.40 to map all SNPs to genomic features. SNPs that occurred within 200 bases from the 5′ or 3′ UTR were considered upstream or downstream effects that possibly correspond to regulatory motifs. If the candidate SNP occurred further than 200 bases from either UTR, it was classified as being intergenic. SNPs with different features attributed to alternative splicing or adjacent/overlapping genes were counted separately for each feature.

We measured the overrepresentation of selected SNPs in a given feature using a chi-square test comparing the counts of selected SNP and nonselected SNP against the remaining selected and nonselected SNPs.

### Gene Ontology analysis

As long genes have a higher probability to contain false-positive candidate SNPs than short genes, gene length bias may result in a spurious overrepresentation of Gene Ontology (GO) categories containing long genes. We therefore tested for an enrichment of GO categories of the 2000 candidate SNPs from the comparisons B-M and B-E using the software Gowinda (Kofler *et al.* in press; http://code.google.com/p/gowinda/). Gowinda performs a permutation test by randomly drawing SNPs without replacement until the number of corresponding random genes equals the number of candidate genes containing candidate SNPs and records GO category hits. This procedure is repeated 10 million times and an empirical distribution of GO category abundance for randomly drawn SNPs is derived for every GO category. The significance of GO category enrichment for the candidate SNPs is directly estimated from this empirical distribution and an empirical FDR correction is used to account for multiple testing. We used the annotation v5.40 of *D. melanogaster*, obtained a GO association file for *D. melanogaster* (flybasecgid_gene_id) from FuncAssociate2 (Berriz *et al.* 2003; http://llama.mshri.on.ca/funcassociate/) and used Gowinda with the following parameters: --simulations 10000000 --gene-definition --unit genes --min-significance 0.000001.

### Other bioinformatic analyses

All other analyses are based on in-house Python and Perl scripts, which are available at Dryad (doi: 10.5061/dryad.60k68).

## Results

We used laboratory natural selection (experimental evolution) by exposing a freshly collected population of *Drosophila melanogaster* in triplicate to a novel environment that consists of laboratory culture conditions in combination with an elevated temperature regime, with daily fluctuations between 18 and 28 °C. Using paired-end Illumina sequencing of pooled individuals (Pool-Seq), we estimated allele frequencies in each of the three replicate populations at three different time points: (i) at the beginning (B) of the experiment (base population); (ii) in the middle (M) of the experiment (generation 15/23); and (iii) at the end (E) of the experiment (generation 37). For one replicate, we also sequenced individuals at generation 27. The average sequence coverage for the genome of the analysed populations ranged from 30 to 64-fold (Table S1, Supporting information).

### Molecular variability in the experimental population

**Fig. 1:**
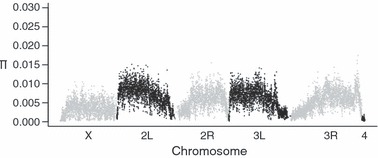
Genome-wide polymorphism pattern in the base population. Estimates of π in non-overlapping 10 kb windows (Kofler *et al.* 2011), plotted against chromosomal position. Consistent with other reports, variability is reduced towards the centromere and telomere, lower on the X chromosome, and extremely low on the 4th chromosome. Since all replicates showed a qualitatively identical pattern, only replicate 1 is shown here.

By statistically identifying consistent AFC among replicates, we focused our analysis on standing genetic variation, thus excluding potential beneficial *de novo* mutations that might have occurred in only one replicate. We found the genetic variability of our base population to be typical for a natural cosmopolitan population (Table S2, Supporting information), with more than 1.6 × 10^6^ SNPs, suggesting the existence of ample genetic variation for selection to act upon. The distribution of variation over the chromosomes also followed the classical pattern, with reduced variability towards the telomeres and centromeres, low variation on the fourth chromosome (Fig. 1) and less variation on the X chromosome than on the autosomes.

The experimental populations were maintained at a census size of about 1000 individuals. Using temporal allele frequency data from our experiment (Bollback *et al.* 2008), we estimated the effective population size to be relatively high, on the order of at least 200 individuals (Fig. S1, Supporting information), which should allow for an efficient response to selection (Weber & Diggins 1990). Interestingly, we found that the loss of heterozygosity was highly heterogeneous among chromosomes. The X chromosome, for which we expected the highest amount of drift because of its smaller effective population size (males have only a single X chromosome), showed the least reduction in heterozygosity. The third chromosome, by contrast, exhibited a substantial loss of heterozygosity (Table S2, Supporting information). This pattern, which is inconsistent with neutral evolution, clearly suggests the existence of strong selection during adaptation to the novel laboratory environment.

### Identification of selected SNPs

**Fig. 2:**
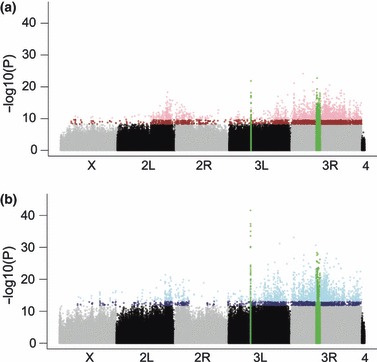
Genome-wide distribution of selected SNPs. Manhattan plots showing (a) *P*-values of all SNPs identified from the comparison between the base and the middle of the experiment (B-M) and (b) *P*-values of all SNPs identified from the comparison between the base and end of the experiment (B-E). SNPs based on the top 0.001%*P*-value threshold from simulations are highlighted in dark red for B-M and dark blue for B-E. The top 2000 candidate SNPs are highlighted in light red for B-M and light blue for B-E. After only 15 generations of adaptation to a novel environment, we detected a large number of selected SNPs that show a more pronounced allele frequency change (AFC) than expected by chance (drift). Note that the distribution of selected SNPs is not homogeneous across the chromosomes. Chromosome *3R* and regions towards the centromere show a highly pronounced overrepresentation of significant SNPs. SNPs highlighted in green are located in two regions that were excluded from the analysis (see Material and methods).

We conservatively assumed that selected alleles would show a strong and consistent change in allele frequency among replicate populations across two time points of the selection experiment and used a CMH test (Agresti 2002), in combination with forward Fisher–Wright simulations, to identify those SNPs that responded to selection rather than to genetic drift. After only 15 generations of evolution in the novel laboratory environment, we were able to identify almost 5000 SNPs that changed their allele frequency across replicates more than expected under drift, using a *P*-value threshold based on the top 0.001% of simulated AFC under drift (genome-wide FDR < 0.005; Fig. 2).

### Two different trajectories of selected SNPs

As the significance levels based on our computer simulations are only approximate because of LD (see Material and methods), we focused on the 2000 most significant SNPs from the comparison between the B-M and B-E populations (FDR < 0.005; Fig. 2). Owing to the small number of significant loci in the M-E comparison, we restricted our analysis to the B-M and B-E contrasts (see Supporting Information for further details). To characterize the dynamics of selected SNPs and to identify potential selective sweeps, we followed the allele frequency trajectories of these top SNPs throughout the entire experiment. Remarkably, we found that the trajectories of the two groups (B-M, B-E) of selected alleles form two qualitatively distinct categories: (i) alleles characterized by a rapid increase in frequency, followed by a phase of little AFC (B-M); and (ii) alleles that exhibit a continuous increase in frequency throughout the entire experiment (B-E).

**Fig. 3:**
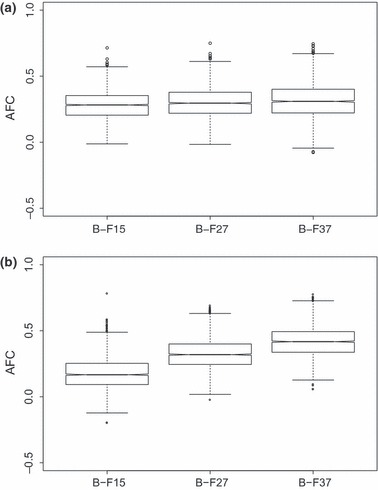
Different trajectories of selected SNPs. We determined the 2000 most significant SNPs at two time points during the laboratory natural selection experiment. The allele frequency trajectories of these SNPs were followed in one replicate by comparing the frequency change of the selected allele at three different time points relative to the beginning of the experiment: B-F15, base population compared with generation 15; B-F27, base population compared with generation 27; and B-F37, base population compared with generation 37. The boxes in the box–whisker plots contain the data between the 25th and 75th percentile; the lower whisker depicts the lowest value within the 1.5 interquartile range (IQR) of the lower quartile, and the upper whisker depicts the highest value within the 1.5 IQR of the upper quartile. The data points outside the whiskers represent outliers. (a) Trajectories of the 2000 most significant SNPs, comparing the base population (B) and middle (M) population (generations 15–23). Initially, the frequencies of selected alleles increase rapidly, but only very slightly during the rest of the experiment. (b) Trajectories of the 2000 most significant SNPs, comparing the base population (B) and the end (E) population (generation 37). Selected SNPs increase continuously in their frequencies throughout the entire experiment. The dynamical behaviour of allele frequency change (AFC) differs significantly between (a) and (b). Note that significant loci were identified from all three replicates, but the trajectory is shown here for only one replicate; trajectories for the other two replicates are shown in the Fig. S2 (Supporting information). Similar results were obtained when using all SNPs instead of the top 2000, with a cut-off based on the top 0.001% of simulated SNPs.

For the first class of alleles, we observed pronounced AFC (median B-M, 0.28) during the first 15 generations, but only very little change (median M-E, 0.027) for the rest of the experiment (Figs 3 and S3, Supporting information). Importantly, this observed plateauing is not an artefact of the pairwise comparison between B and M because neutral simulations did not exhibit this pattern (see Table S7, Supporting information). Moreover, the plateau in AFC is not readily explained by fixation of selected alleles because only 9% of these candidate SNPs had a frequency higher than 0.90 at generation 37. Finally, the plateau is unlikely an artefact of neutral SNPs linked to a few selected SNPs (see ‘Independence of SNPs’ in Supporting Information).

In contrast, for the second class of alleles, the frequency trajectories of the 2000 most significant SNPs from the comparison between the B and E (generation 37) populations behaved markedly differently (see Supporting Information for a formal test). Consistent with a simple additive model of gene action, these loci experienced continuous AFC (median B-E, 0.42) across the entire experiment (Figs 3, S3 and S12, Table S7, Supporting information). Notably, this set of alleles showed more extreme AFC at generation 37 than the candidates from the comparison B-M, while the opposite was true for the middle of the experiment (Fig. 3). Together, these observations strongly suggest that the two classes of alleles are governed by very distinct frequency dynamics. We only found a relatively small number of loci that were shared between the two comparisons B-M and B-E (447 out of 2000 SNPs, or 22%).

A classical prediction of evolutionary genetics is that adaptation is driven by beneficial mutations that increase in frequency until they become fixed. However, among the 2000 most significant SNPs from both comparisons B-M and B-E, we very rarely observed fixation (frequency ≥ 0.99) of selected alleles. After 37 generations of experimental evolution, only 2.1% of the SNPs from the B-M comparison were fixed, whereas for the B-E comparison only 0.5% of the SNPs showed fixation. Thus, while we found a rapid and pervasive genomic response to selection, we clearly failed to detect strong signatures of extensive selective sweeps in our experimental evolution experiment.

### Genomic distribution of selected SNPs

The identified candidate SNPs were not distributed homogenously across chromosomes. Although 11% of all SNPs were located on the X chromosome, only 0.3% (B-M) and 0.9% (B-E) of all significant SNPs were found on this chromosome (Table S3, Supporting information). In contrast, we found that significant SNPs were highly overrepresented on the right arm of the third chromosome (*3R*). As *3R* carries the cosmopolitan inversion *In*(*3R)P*, which is thought to play a major role in latitudinal (thermal) adaptation (Anderson *et al.* 2003; De Jong & Bochdanovits 2003), we tested whether the high number of selected SNPs on *3R* could be explained by a change in inversion frequency. However, when comparing the number of SNPs between the region of the chromosome arm spanned by the inversion and the inversion-free part of *3R*, we failed to find an excess of candidate SNPs within the inversion (Table S4, Supporting information). Moreover, the inversion frequency decreased during the laboratory natural experiment from around 11% in the base population to about 1% in generation 38 (Table S5, Supporting information). Thus, the allele frequencies of significant SNPs changed about twice as much as the inversion frequency. Furthermore, the shape of the AFC trajectories for B-M and B-E did not change when SNPs located inside the inversion were excluded from the analysis (Fig. S10, Supporting information). The direction of the inversion frequency change observed in our experiment also contrasts with the pattern found in natural populations, where inversion frequency is positively correlated with environmental temperature (Mettler & Voelker 1977; Anderson *et al.* 2005). Together, these data suggest that *In*(*3R)P* is unlikely to be the cause of the high number of candidate SNPs on *3R*.

### Characterization of selected SNPs

As an over- or under-representation of candidate SNPs relative to the entirety of SNPs might provide important insights into the basis of adaptation, we next mapped the significant SNPs to the annotated gene features of *D. melanogaster* (Table S6, Supporting information). Interestingly in the B-M contrast, candidates were significantly enriched in introns and in nonsynonymous exonic positions (Table S6, Supporting information). Moreover, for some gene feature categories, we also observed significant under-representation of candidate SNPs. In the B-M comparison, intergenic regions harboured fewer candidate SNPS than expected. We also noted a highly significant deficiency of candidate SNPs for synonymous SNPs in coding sequences (Table S6, Supporting information). We reason that this under-representation might reflect an enrichment of selected SNPs among the other classes. In the case of the B-E contrast, we observed a significant overrepresentation of selected SNPs in 3' UTR, downstream, and intronic regions.

To further functionally characterize our set of candidate SNPs, we performed GO analysis on all genes containing significant SNPs. On the basis of a FDR cut-off ≤ 0.1, the comparison B-M revealed a strong enrichment of genes involved in DNA packaging, DNA condensation, DNA conformation change, catalytic activity and metabolic processes. This latter category was also marginally significant in the comparison B-E (Table S8, Supporting information).

### Low levels of linkage disequilibrium permit pinpointing of selected alleles

Because natural populations of *D. melanogaster* are known to exhibit rather low levels of linkage (e.g. Miyashita & Langley 1988), and as our data are based on pooled individuals without information of haplotype structure, our analyses ignored linkage. Although the build-up of LD has been intensively studied in selection experiments (Bulmer 1976; Hospital & Chevalet 1996), it is presently unclear whether and how drift and selection might have affected the haplotype structure in our experimental populations over time.

**Fig. 4:**
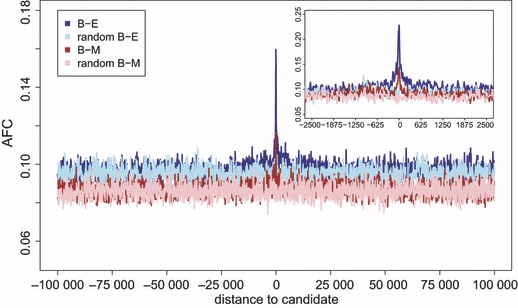
Decay of significance around significant SNPs. Median allele frequency changes (AFC) between base and generation 37 across all replicates for SNPs grouped into 100-bp windows flanking the 2000 most significant SNPs from the comparisons B-M (red) and B-E (blue). The median of 2000 position-adjusted random SNPs is shown in light red (B-M) and light blue (B-E). Position adjustment is necessary because the recombinational environment differs along the chromosomes, and we noticed some heterogeneity in genetic drift among the chromosomes. To minimize possible linkage disequilibrium (LD) with selected SNPs, position-adjusted SNPs were picked 500 kb upstream of each of the 2000 selected SNPs. The pronounced drop in *P*-values suggests that there is no strong LD between selected SNPs and their flanking sites. The inset shows a blow-up of the genomic region around the candidate SNPs based on 50-bp bins. Note that as this graph does not include the 2000 significant SNPs, the peak of *P*-values can only be caused by linked SNPs. A corresponding figure showing the Cochran–Mantel–Haenszel (CMH) *P*-value is given in the Fig. S8 (Kofler & Schlötterer 2012, in press).

While Pool-Seq is an excellent approach for estimating allele frequencies (Futschik & Schlötterer 2010), it does not permit direct inferences about haplotypes, and we were thus unable to directly determine if LD increased the number of significant SNPs in our data set. However, we reasoned that – if LD indeed has a major effect on SNP number – it would increase the significance of SNPs flanking our candidate SNPs. To test this notion, we plotted the median AFC from the base to generation 37 in 50- and 100-bp windows around the candidate SNPs and consistently observed a pronounced decay of AFC around our candidate SNPs (Fig. 4). This rapid decay in flanking regions suggests that the influence of linkage is limited. This is also confirmed by a clear-cut overrepresentation of significant SNPs in introns.

## Discussion

Our data show that already after 15 generations, a strong genomic response to selection can be observed. Remarkably, those SNPs that were most strongly affected by selection during the first generations did not continue to change their frequency at the same rate but appeared to plateau.

Our findings at the level of allele frequencies clearly resemble classical observations on phenotypic responses to artificial selection (Mather & Harrison 1949; Clayton & Robertson 1957; Gilligan & Frankham 2003). Although the available evidence suggests that a loss of additive genetic variation can limit the response to selection (Robertson 1960; Brown & Bell 1961), several reverse selection (Scowcroft 1966; Teotonio & Rose 2000) and chromosome extraction experiments (Brown & Bell 1961) indicate that selected traits often harbour significant amounts of (nonadditive) genetic variation despite having ceased to respond to selection. Because in our experiment most of the selected alleles were not fixed (and either plateaued or continued to increase in frequency), we can rule out that the loss of additive genetic variation has imposed a limit upon the response to selection. It is interesting to note in this context that a recent study of a long-term selection experiment has also failed to observe the fixation of selected alleles (Burke *et al.* 2010). On the basis of a model by Chevin & Hospital (2008), these authors suggest that, as selection coefficients may decrease when a polygenic quantitative trait approaches its optimum, changes in allele frequencies may slow down and level off without reaching fixation. While it is possible that this model also accounts for the frequency plateaus seen in our experiment, it cannot readily explain our observation of alleles that continued to rise in frequency. Nevertheless, if the two classes of alleles in our experiment would affect distinct traits, with the continuously rising alleles affecting other traits with more distant optima than those that plateau, the model by Chevin & Hospital (2008) might still apply to our data. Potential alternative explanations for allele frequency plateaus might include dominance, sign epistasis or antagonistic pleiotropy, but we cannot presently distinguish among these possibilities. It will thus be of major interest to determine the proximate causes of allele frequency plateaus in future work.

Interestingly, an experimental evolution experiment using a pool of recombinants from two yeast strains also found plateauing of allele frequencies. As this plateauing was only seen for haploids, but not for diploids, dominance seems to be an unlikely explanation (Parts *et al.* 2011). Furthermore, despite the fact that candidate SNPs quickly raised in frequency (<15 generations), the majority of the plateauing alleles only reached intermediate frequencies (Fig. S6, Supporting information). This argues strongly against dominance as being the cause of the plateau. Given that our novel laboratory environment involved fluctuating temperature regimes, we favour antagonistic pleiotropy and overdominance as the most parsimonious explanations for the plateauing of selected SNPs.

Our experiment was designed to study the genomic response owing to the adaptation to a novel environment. This novel environment consisted of laboratory culture conditions at an elevated temperature. It is therefore interesting to compare our results to previous studies on temperature stress. For example, we did not detect any candidate genes involved in the resistance to heat shock (Hoffmann *et al.* 2003; Morgan & Mackay 2006). Our interpretation of this discrepancy is that the heat stress in our experiment differs qualitatively from the heat shock applied in other studies and that different pathways might be involved. We anticipate that the comparison of our results to natural populations from habitats with different temperatures will shed further light on the question to what extent laboratory adaptation to temperature reflects selection in the wild.

Our work clearly demonstrates the power of combining laboratory natural selection with next generation sequencing for studying the genomic response to adaptation to a novel environment. Consistent with the few genome-wide studies of selection in experimental populations available to date, we have identified a large number of candidate SNPs that have responded to selection. However, in contrast to these studies, here we have directly compared the evolved populations with their ancestral base population and analysed the temporal dynamics of AFC across different time points during the experiment. Our data suggest that selected alleles can exhibit dramatically different dynamical trajectories, an observation that is clearly at odds with simple models of adaptation that predict a continuous increase in the frequency of beneficial alleles followed by fixation.

In particular, as we have identified different and largely nonoverlapping sets of selected SNPs at different time points during our experiment, our study casts doubt on whether it will be easily possible to understand the process of adaptation for complex traits from the analysis of natural populations without temporal sampling. Clearly, future laboratory natural selection experiments with denser temporal sampling over a longer time and including information on haplotype structure and LD dynamics will provide major insights into how many replicates are required for unambiguously mapping causative alleles. We anticipate that over the next few years experimental evolution studies combined with the latest sequencing technologies will prove invaluable for a better understanding of the evolutionary and functional basis of adaptation (Rose *et al.* 2011).

## Data accessibility

The raw FASTQ files for all populations are available from the European Sequence Read Archive (http://www.ebi.ac.uk/ena/about/search_and_browse) under the Accession no. ERP001290.

SNP data sets (available in VCF file format or a synchronized pileup files) as well as a collection of custom written Python scripts are available from the Dryad database (http://datadryad.org) under the accession: doi: 10.5061/dryad.60k68.

## Supporting information

**Appendix S1** Methods.

**Fig. S1** Estimation of effective population size from temporal allele frequency data.

**Fig. S2** Different trajectories of selected SNPs.

**Fig. S3** Allele frequency trajectories for all SNPs.

**Fig. S4** Frequency distribution of the slopes of allele frequency changes for the 2000 most significant SNPs identified in the comparisons B-M and B-E.

**Fig. S5** Frequency distribution of the slopes of allele frequency changes for significant SNPs identified in the comparisons B-M and B-E.

**Fig. S6** Allele frequencies of selected SNPs.

**Fig. S7** Allele frequency changes for significant SNPs identified in the comparisons B-M.

**Fig. S8** Decay of *P*-values around B-M candidate SNPs.

**Fig. S9** Allele frequency changes of subsets of the candidates separated by a large distance.

**Fig. S10** Allele frequency changes of subsets of the candidates located outside of the inversion *In(3R)P*.

**Fig. S11** Manhattan plots of average allele frequency changes and cumulative coverage.

**Fig. S12** Distance between neighboring candidate SNPs.

**Fig. S13** CMH *P*-value distribution of sequencing and simulated data.

**Fig. S14** AFC trajectories of single SNPs.

**Table S1** Coverage for each population.

**Table S2** *π* estimates for each chromosome arm and population (replicate 3).

**Table S3** Distribution of all SNPs and the 2000 most significant SNPS for the comparison B-M and B-E.

**Table S4** Distribution of all SNPs and the 2000 most significant SNPS on chromosome 3R for the comparison B-M and B-E.

**Table S5** Frequency of the inversion *In(3R)Payne* determined by PCR.

**Table S6** Distribution of all SNPs and the 2000 most significant SNPs split by genomic feature for the comparison B-M and B-E.

**Table S7** Median allele frequency changes of all pairwise population comparisons in replicate one of the top 2000 candidates from the cage experiment and the top 2000 candidates of the simulated data.

**Table S8** Table of overrepresented GO Terms withan FDR ≤ 0.4 for datasets B-M and B-E.
