# Supplementary material for: Adaptation of Drosophila to a novel laboratory environment reveals temporally heterogeneous trajectories of selected alleles
Source: Mol Ecol. 2012 Oct;21(20):4931–41. doi: 10.1111/j.1365-294X.2012.05673.x (PMC3533796; doi:10.1111/j.1365-294X.2012.05673.x)
Supplement: Supplementary file 2 [file mec0021-4931-SD2.pdf]

Supplementary Information

Independence of SNPs 2
Validation of two different selection trajectories 4
Recessive deleterious alleles 5
Plateaus in the B-M comparison are unlikely an artifact of hitchhiked, non-selected SNPs 6
Exclusion of the M-E comparison 8
References 8
Supplementary Figures and Tables 9

Independence of SNPs

One simplification of our statistical analysis for the identification of selected SNPs was the assumption of their independence. The drawback of this approach is that linkage disequilibrium might result in the false classification of neutral SNPs as selected ones. Consequently, this may cause the clustering of SNPs that are classified as selected SNPs. Increased LD should thus be manifested as more pronounced clustering, eventually leading to large blocks of highly correlated SNPs. Furthermore, with an increasing number of neutral SNPs being wrongly classified as selected (due to LD), no functional class (e.g., introns) would be expected to be overrepresented. To study the impact of our assumption of independence of SNPs we performed three analyses. First, we evaluated to what extent SNPs in the proximity of those classified as being selected show evidence of non-neutral behavior (Fig. 4 and Supplementary Fig. 8). Importantly, while SNPs in the proximity of our candidate SNPs also showed some indication of selection, this effect very quickly leveled off within a few hundred bases. Therefore only a tiny proportion of the genome is affected by linkage. Second, we asked whether functional categories were overrepresented among the SNPs classified as being selected. In the presence of large blocks of correlated SNPs, the pattern of random SNPs should not differ from that of selected SNPs. We found, however, that a larger number of selected SNPs was located in introns and non-synonymous codon positions than expected by chance (i.e., the fraction of non-selected SNPs in introns and non-synonymous codon positions was lower than that of selected SNPs; see Supplementary Table 6). Third, we measured the distance between neighboring candidate SNPs and plotted the distribution of

distances. Although there is a slight excess of neighboring candidates within a 100 bp distance (14.8% for B-M and 17.1% for B-E; see Supplementary Fig. 12), which is in concordance with the results shown in Fig. 4, the vast majority of candidates is separated by much larger distances: over 40% of all top 2000 SNPs are located (43.8% for B-M and 40.1% for B-E) more than 10 kbp away from the closest neighbor. In summary, these analyses show that the majority of SNPs classified by us as being selected very likely represent true targets of selection and that the number of false positives due to our simplifying assumption is not very large.

The absence of large blocks of correlated SNPs may appear surprising, in particular in the light of previous experimental evolution studies. Nevertheless, the explanation for this pattern is quite straightforward: contrary to other studies, we started from a freshly collected outbred population and monitored adaptation over a very short time scale. Selected SNPs were thus already segregating at a moderate allele frequency at the beginning of our experiment (see Supplementary Fig. 6). Given the low levels of LD in natural *D. melanogaster* populations (Miyashita & Langley 1988), favorable alleles occur in many different haplotypes. Hence, if selection increases the frequency of a favorable allele, the influence on linked sites is only moderate (as seen in Fig. 4). Also note that a different pattern is expected for selection of novel beneficial mutations, leading to large block of correlated sites; however, only few recombination events occurred during our experiment due to the small number of generations.

Importantly, the extent to which our assumption of independence among SNPs is met depends on the amount of LD present in natural *D. melanogaster* populations. While the genomic levels of LD in natural *D. melanogaster* populations are not yet known, some predictions can be made based on the recombination map: low levels of recombination on the 4th chromosome and towards the telomeres and centromeres are expected to result in higher levels of LD (and hence a higher false positive rate based on our test).

An alternative to our assumption of independence would be to perform computer simulations that incorporate levels of LD in the base population, recombination rates and selection. Given that neither the patterns of LD across the *D. melanogaster* genome are known nor the details of the selection process are understood, we do not think that simulations would be a more powerful approach for analyzing genome-wide patterns of allele frequency changes in our experiment.

Validation of two different selection trajectories

We further validated the trajectories of selected SNPs identified in the comparison B-M or B-E by comparing the slopes of the trajectories (allele frequency changes) for the comparisons of B to generation 15, B to generation 27, and B to generation 37 for the replicate population for which a total of four time points was sequenced. As expected with two different classes of allele frequency trajectories, we found two distinct distributions of slopes of AFC relative to the base population that differed significantly from each other

(Wilcoxon Rank Test, W=625559, P<0.001; see Supplementary Fig. 4). For candidate SNPs from the comparison B-M the median of the slope was close to zero, confirming the existence of an allele frequency plateau. In contrast, for the comparison candidate SNPs from B-E, we found a positive slope consistent with the notion that allele frequencies increase steadily. Because the identification of significant SNPs may result in biased estimates of allele frequency change, we repeated the analysis by comparing (i) B to generation 27 and to generation 37 for those SNPs that were significant in the comparison B-M and (ii) B to generation 15 and to generation 27 for those SNPs that were significant in the comparison B-E. As expected, this analysis generated a broader distribution of slopes, but the overall pattern of two distinct classes of allele frequency trajectories was confirmed (see Supplementary Fig. 5).

Recessive deleterious alleles

A classic result from *Drosophila* population genetics is the absence of recessive lethals on the *X*-chromosome. Since we noticed that selected alleles were substantially underrepresented on the *X*-chromosome, this might suggest that recessive deleterious alleles are responsible for the observed allele frequency changes. Nevertheless, we do not think that this is likely to be the case. While recessive lethal alleles are abundant in *Drosophila*, the frequency of each allele is low (Dobzhansky & Wright 1941). Thus, even if the population size is dramatically reduced (e.g., as in our experiment, N_e of at least 200), only very rarely will individuals be homozygous for a recessive deleterious allele. Rare

recessive deleterious alleles are therefore very unlikely to cause the kind of major allele frequency changes we have observed in our experiment.

Plateaus in the B-M comparison are unlikely an artifact of hitchhiked, non-selected SNPs

Two – not mutually exclusive – scenarios can explain how linkage between neutral and selected SNPs could potentially lead to allele frequency plateaus. Both scenarios assume that linked neutral SNPs substantially outnumber selected SNPs.

In the first scenario, neutral SNPs are only partially linked to selected ones. During the experiment selected SNPs become fixed, but partially linked neutral SNPs will reach a plateau. The level of the plateau depends on the extent of LD between linked SNPs at the beginning of the experiment. We consider this explanation unlikely since the significant SNPs in the B-M comparison experienced a large allele frequency change from the beginning to the middle of the experiment. If these SNPs were partially linked to a causative SNP, the causative SNPs would have undergone an even more extreme allele frequency shift than the majority of unselected SNPs. To test whether this hypothesis is consistent with our data, we analyzed the frequency changes of SNPs with a frequency of at least 0.9 at the middle of the experiment (M) (Supplementary Figure 8). As expected, these SNPs changed significantly less in frequency than the full data set of the 2000 SNPs from the B-M comparison (Wilcoxon Rank test:

W= 104068, P<0.001). In total, only two SNPs changed from B to M more than 40%.

The second scenario assumes plateauing of linked neutral SNPs through recombination during the spread of the selected SNP(s). Again, we consider this scenario to be unlikely since probability of recombination with a non-selected chromosome is highest at the onset of the experiment. Thus, as in the first scenario above, the selected allele must change more in frequency than the neutral linked allele. Since recombination scales with distance from the selected site, we asked whether for the 2000 SNPs identified in the comparison B-M we find a decrease in allele frequency change with distance from a putatively selected SNP by comparing the middle (M) of the experiment with the end (E). The comparison B-M indicated that only a very narrow region of about 600bp shows elevated allele frequency change (Figure 4 and Supplementary Figure 8). This graph clearly suggests that the effect of hitchhiking is probably restricted to a very narrow region. Moreover, we did not observe a reduction of the size of this window as compared to generation 37 (Supplementary Fig. 8). This scenario can therefore also not readily explain the plateauing observed in our experiment.

Furthermore, we reduced the 2000 candidate SNPs of the comparison B-M to the most significant SNPs with a minimal physical of 20 and 50 kilo-base-pair distance to each other (Supplementary Fig. 9) to minimize the possibility of hitchhiking SNPs contributing to the pattern of the trajectory. The results for both reduced datasets are very similar to the full dataset (Figure 3), which further suggests that the trajectories are not a result of linkage disequilibrium.

Exclusion of the M-E comparison

In addition to the comparisons B-M and B-E discussed in the manuscript, we also analyzed the third comparison between M and E. We determined the top candidates based on the *P*-value cutoff from the top 0.001% simulated SNPs. In contrast to the 4822 and 3490 candidates found for B-M and B-E respectively, the M-E comparison only yielded 391 candidates. Additionally, empirical FDR calculated from the simulated data (see Material and Methods for a description) resulted in a more than 10-fold higher FDR (>0.05) for M-E as compared to the other comparisons (B-E and B-M; both <0.005). Since we cannot exclude the possibility of a large excess of false positives in this dataset we excluded the M-E dataset from all further analyses,

References

Bollback JP, York TL, Nielsen R (2008) Estimation of 2Nes from temporal allele frequency data. *Genetics* **179**, 497-502.

Dobzhansky T, Wright S (1941) Genetics of Natural Populations. V. Relations between mutation rate and accumulation of lethals in populations of *Drosophila pseudoobscura*. *Genetics* **26**, 23-51.

Miyashita N, Langley CH (1988) Molecular and phenotypic variation of the *white* locus region in *Drosophila melanogaster*. *Genetics* **120**, 199-212.

Supplementary Figures and Tables

Supplementary Fig. 1. Estimation of effective population size from temporal allele frequency data. The likelihood of different effective population sizes was estimated from 1000 SNPs from each major chromosome arm following the approach of (Bollback *et al.* 2008). The two panels show the estimates for two of replicates. While the likelihood surface is quite flat for most chromosome arms, it is apparent that the effective population size is larger than 200, independent of the dataset used for the estimates.

Supplementary Fig. 2. Different trajectories of selected SNPs. We determined the 2000 most significant SNPs at two time points during the laboratory natural selection experiment. The allele frequency trajectories of these SNPs were followed for each replicate population separately by comparing the frequency change of the selected allele at two different time points (generations 15 and 37) relative to the beginning of the experiment (the graphs show the two replicates for which no data are shown in the main document). (A), (B) Trajectories of the 2000 most significant SNPs in the comparison between base population (B) and middle (M) population (generations 15-23) for two of the three replicates. There is a rapid increase in frequency of the selected allele, but only a very slight increase during the rest of the experiment. (C) and (D) Trajectories of the 2000 most significant SNPs in the comparison base population (B) against end (E) population (generation 37) for two of the three replicates. These SNPs show a continuous increase in frequency throughout the

entire experiment. Thus, there are clearly two distinct classes of alleles that exhibit different dynamics in allele frequency change (AFC) over time. Note that the significant SNPs were identified from all three replicates, but for greater clarity only the trajectories for two replicates are shown here.
We observe a high congruence among the three replicate populations.

Supplementary Fig. 3. Allele frequency trajectories for all SNPs. Analogous to Fig. 3 in the main text, but for all candidate SNPs (cut-off based on top 0.001% of the simulated SNPs) rather than only the 2000 significant SNPs.

Supplementary Fig. 4 Frequency distribution of the slopes of allele frequency changes for the 2000 most significant SNPs identified in the comparisons B-M and B-E. For every significant SNP we calculated the slope for three measurements of AFC in one replicate: base-generation 15, base-generation 27, base-generation 37. The red bars show the frequency distribution obtained from those SNPs that were significant in the comparison B-M; the blue bars show the frequency distribution obtained from those SNPs that were significant in the comparison. B-E. As expected if allele frequencies plateau, the mode of the slopes from the B-M data set is around zero. In contrast, the slope is positive for those SNPs that are significant in the comparison B-E (blue), suggesting that these SNPs show a steady increase in frequency. The two distributions are significantly different (Wilcoxon rank sum test: W = 625559.5, *P*-value < 0.0001). Note for clarity that the slopes shown here are based on a single replicate only. The other replicates showed qualitatively identical patterns (data not shown).

Supplementary Fig. 5 Frequency distribution of the slopes of allele frequency changes for significant SNPs identified in the comparisons B-M and B-E. We repeated the same kind of analysis as shown in Supplementary Fig. 4 but now only using those time point comparisons for B-M and B-E that were not used for identifying candidate SNPs with the CMH test: thus, we calculated the AFC slopes for (i) base-generation 27 and base-generation 37 for all those SNPs found to be significant in the comparison B-M (i.e., base-generation 15) and (ii) base-generation 15 and base-generation 27 for the significant SNPs from the comparison B-E (i.e., base-generation 37). This analysis therefore excludes the actual comparisons used for identifying candidate SNPs in B-M and B-E and might thus avoid potential bias caused by conditioning the analysis on the most significant AFC. As expected, this procedure generated a broader distribution of slopes as compared to those in Supplementary Fig. 4, but statistically confirms the overall distinction between the two classes of trajectories (Wilcoxon rank sum test: W = 943253.5, P-value < 0.0001).

Supplementary Fig. 6 Allele frequencies of selected SNPs. (A) Box plots showing allele frequencies of the 2000 SNPs most significant SNPs in the comparison between the start and the middle of the experiment (B-M) in replicate one. (B) shows the 2000 most significant candidates for the comparison between the start and the end of the experiment (B-E) of replicate one. Note, that for this replicate an additional population at generation 27 was sequenced, which has been included in the calculations for this graph. (C) and (D) show the analogous datasets for replicate two. For this replicate, generation 23 was

sequenced at the middle of the experiment. (E) and (F) show the same analyses for replicate three.

Supplementary Fig. 7 Allele frequency changes for significant SNPs identified in the comparisons B-M. The 2000 most significant SNPs identified in the comparison of B-M were separately analyzed for SNPs with the selected allele being alleles close to fixation ($\geq$0.9 , blue) and SNPs not close to fixation ($<$0.9, light blue). SNPs with the selected alleles being close to fixation in the middle of the experiment (M) experienced smaller allele frequency changes from the base population (B) to the middle of the experiment (M) than the remaining selected SNPs.

Supplementary Fig. 8 Decay of allele frequency changes around B-M candidate SNPs. Median CMH *P*-Values of SNPs flanking the 2000 most significant SNPs of the comparison B-M (red) and B-E (blue) grouped into 100 bp windows (as in Fig. 4). The median of 2000 position-adjusted random SNPs is shown in light red (B-M) and light blue (B-E). The position adjustment is needed since the recombination environment differs along the chromosomes and we noted some heterogeneity in genetic drift among the chromosomes. To minimize possible LD with selected SNPs, position-adjusted SNPs were selected 500 kb upstream of each of the 2000 selected SNPs. The inset is a blow up of the genomic region around the candidate SNPs calculated in 50 bp windows. Note, the most significant SNPs are excluded from this analysis.

Supplementary Fig. 9 Allele frequency changes of subsets of the candidates separated by a large distance.

We chose the most significant SNPs from the top 2000 candidates of the comparison B-M and filtered for SNPs with a minimum distance of 20 (A) and 50 (B) kilo base pairs between them to minimize the possibility of linkage disequilibrium between the remaining candidates. AFC was calculated and plotted similarly to Figure 3.

Supplementary Fig. 10 Allele frequency changes of subsets of the candidates located outside of the inversion *In(3R)P*.

We subtracted the fraction of the top 2000 candidate SNPs not located within the region of the *In(3R)P* inversion for B-M (A) and B-E (B) and calculated the AFC according to the methods in Figure 3.

Supplementary Fig. 11 Manhattan plots of average allele frequency changes and cumulative coverage. Manhattan plots showing the average AFC across all three replicates for all SNPs between base and generation 37 for the comparison B-M (A) and the base and generation 37 for the comparison B-E (B). (C) and (D) show the cumulative SNP-wise coverages of all six populations used for the CMH tests of comparisons B-M and B-E respectively. Note that the highlighted top 2000 candidates are based on CMH tests. The region around the chorion genes on *3L* and the 1 mb region on *3R*, which were excluded from the analyses (see Material and Methods), are highlighted in green. Candidate SNPs are not enriched in regions with an unusual high coverage.

Supplementary Fig. 12 Distance between neighboring candidate SNPs. For every candidate SNP, we calculated the distance in basepairs to the closest neighbor on the same chromosome and plotted the histogram of the distances in 100 bp bins. The distribution was truncated so that it only shows the distribution of neighboring candidate SNPs up to a distance of 10 kb. Note that more than 40% of all candidates from B-M (red) and B-E (blue) are located further than 10 kb away from their nearest neighbor.

Supplementary Fig. 13 CMH *P*-value distribution of sequencing and simulated data. Relative frequencies of CMH *P*-Values for all SNPs from the sequencing and simulated data plotted in 100 bins. Intermediate *P*-values of all datasets are uniformly distributed. However, there is an excess of highly significant SNPs in the B-M and the B-E comparisons for the real data, an effect that is much stronger than for the corresponding simulated data. Note that the leveling-off of the *P*-value distribution towards 1 might be a result of the coverage thresholds used for SNP calling.

Supplementary Fig. 14 AFC trajectories of single SNPs. AFC of candidate SNPs for the comparisons B-M (A) and B-E (B) for replicate 1.

Fig. 1

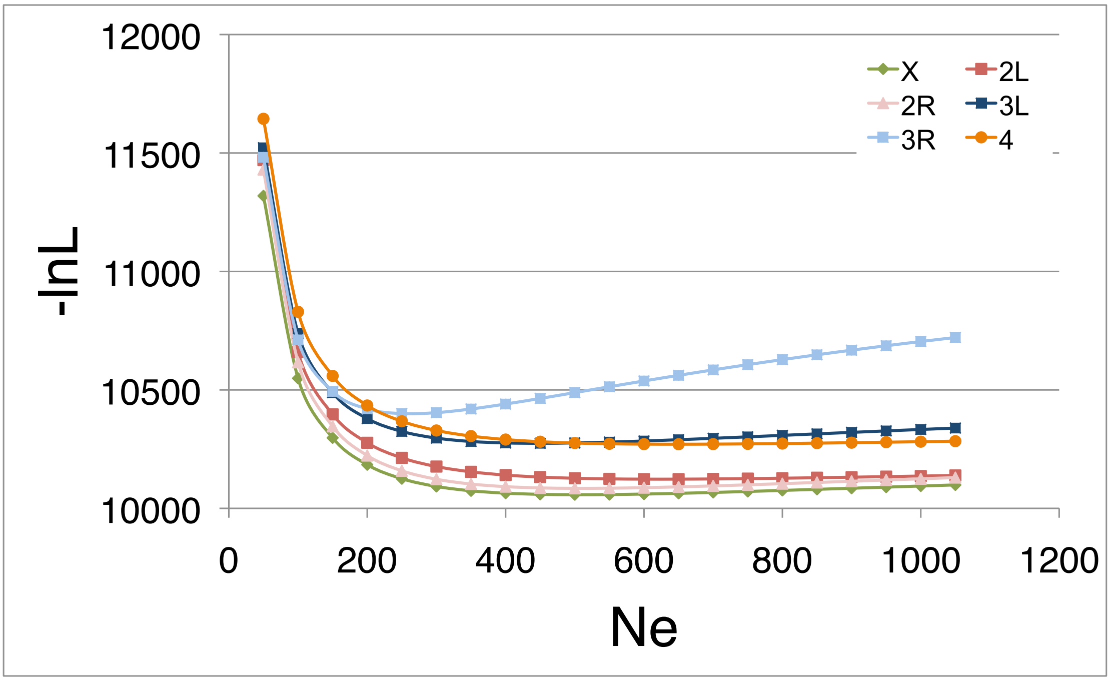
12000
11500
11000
10500
10000
-lnL
0
200
400
600
800
1000
1200
Ne
X
2L
2R
3L
3R
4

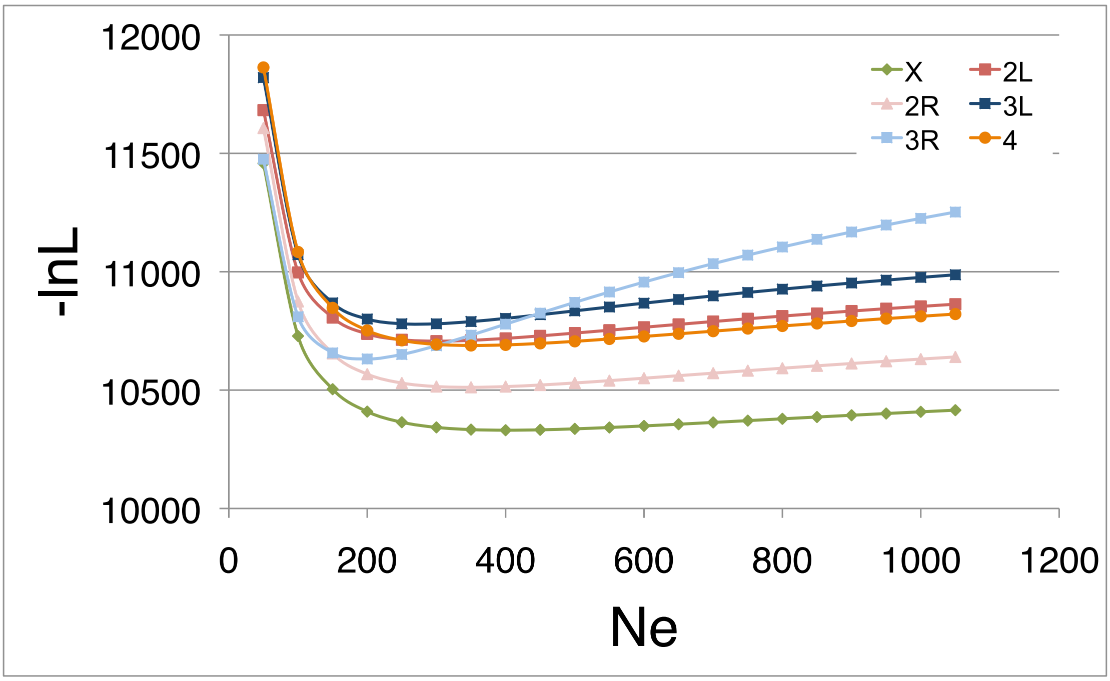
12000
11500
11000
10500
10000
-lnL
0
200
400
600
800
1000
1200
Ne
X
2L
2R
3L
3R
4

Fig. 2

A)

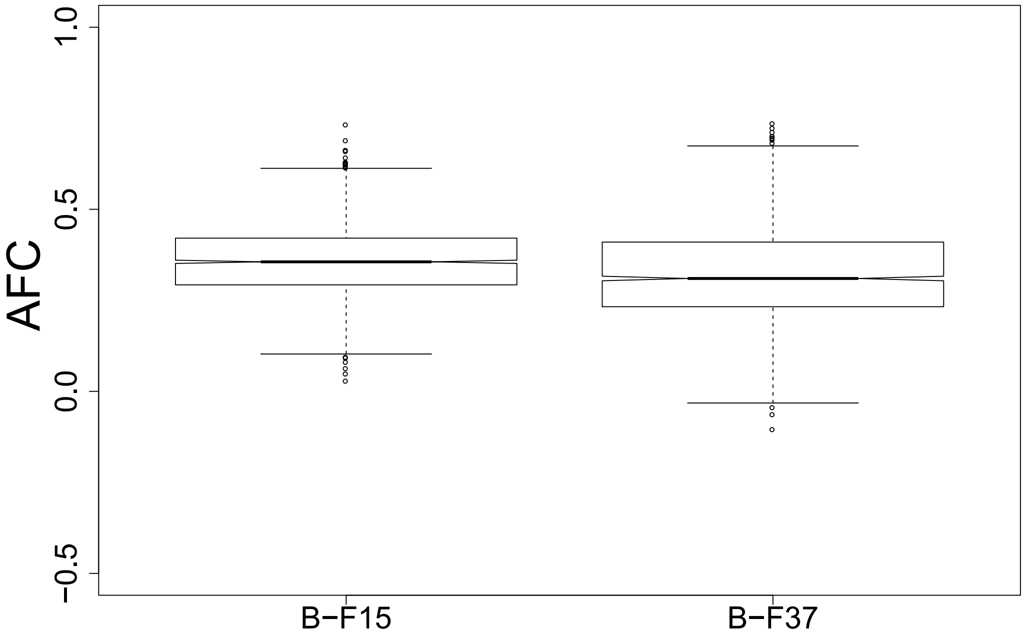

B)

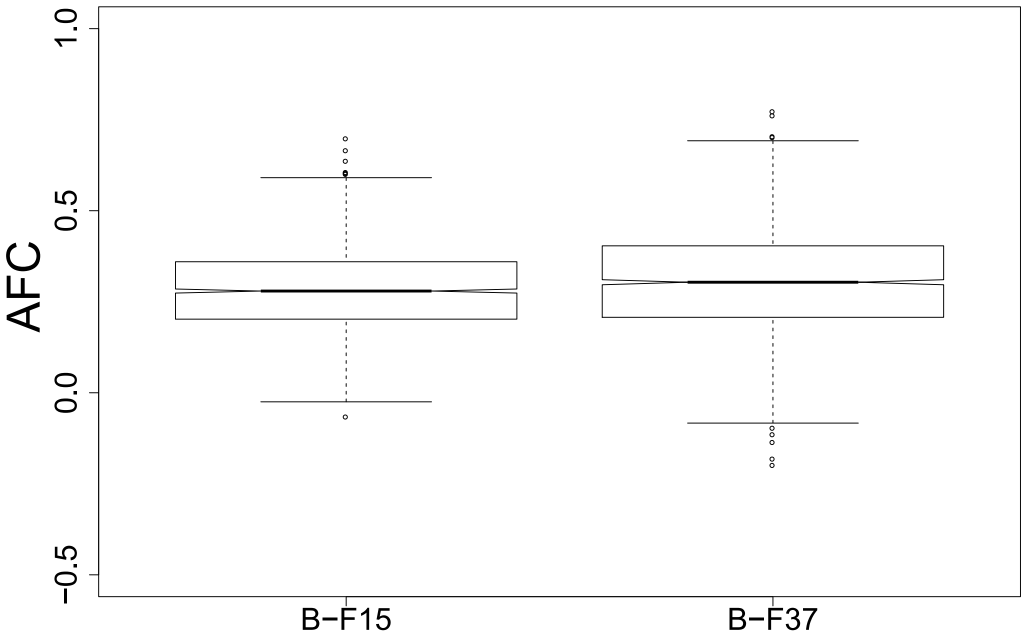
AFC
1.0
0.5
0.0
−0.5
B−F15
B−F37

C)

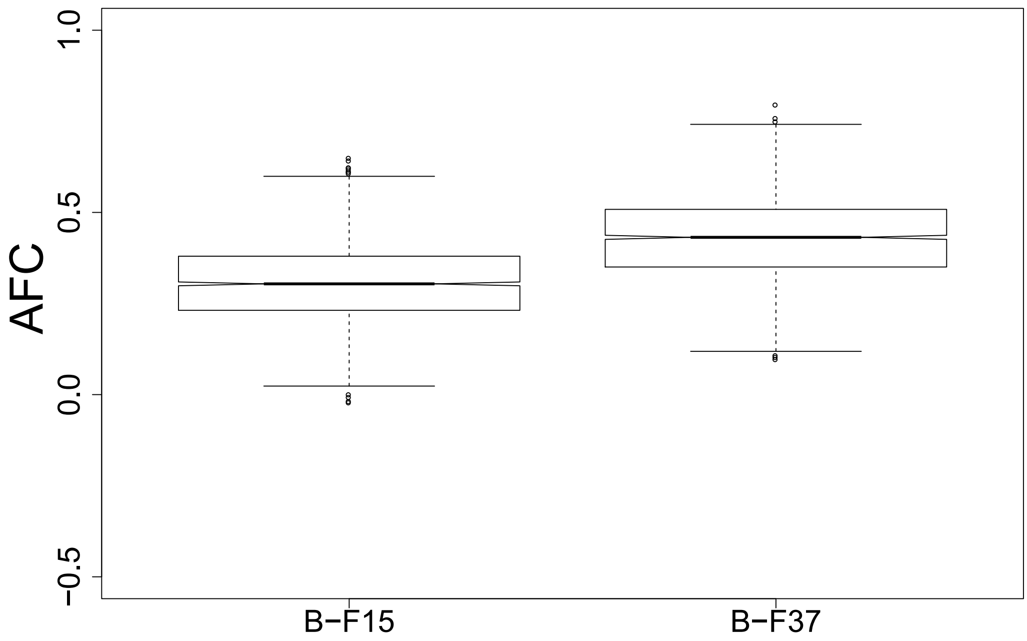

D)

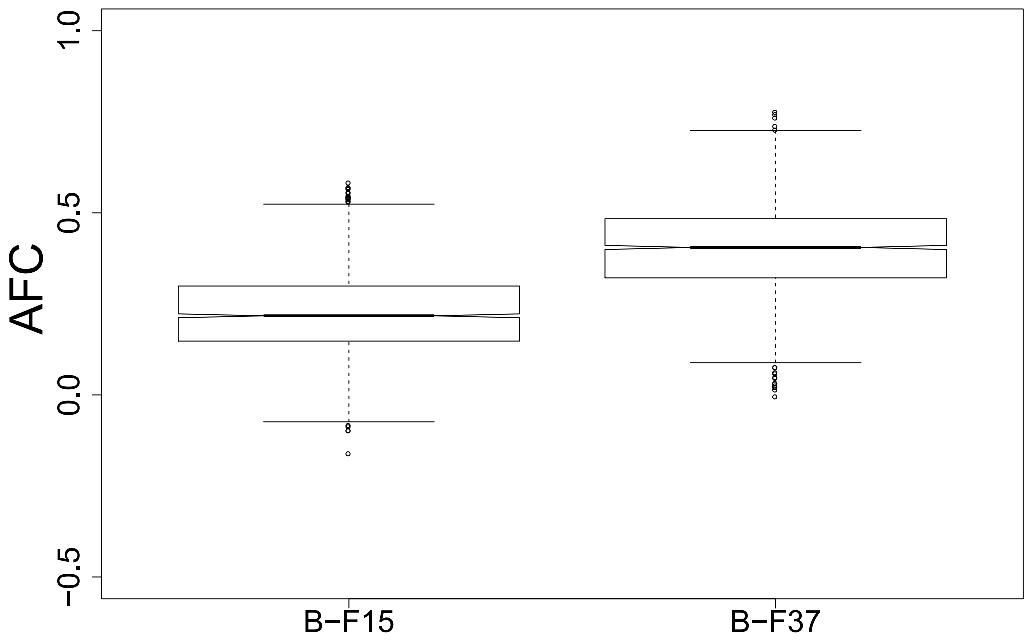

Fig. 3

A)

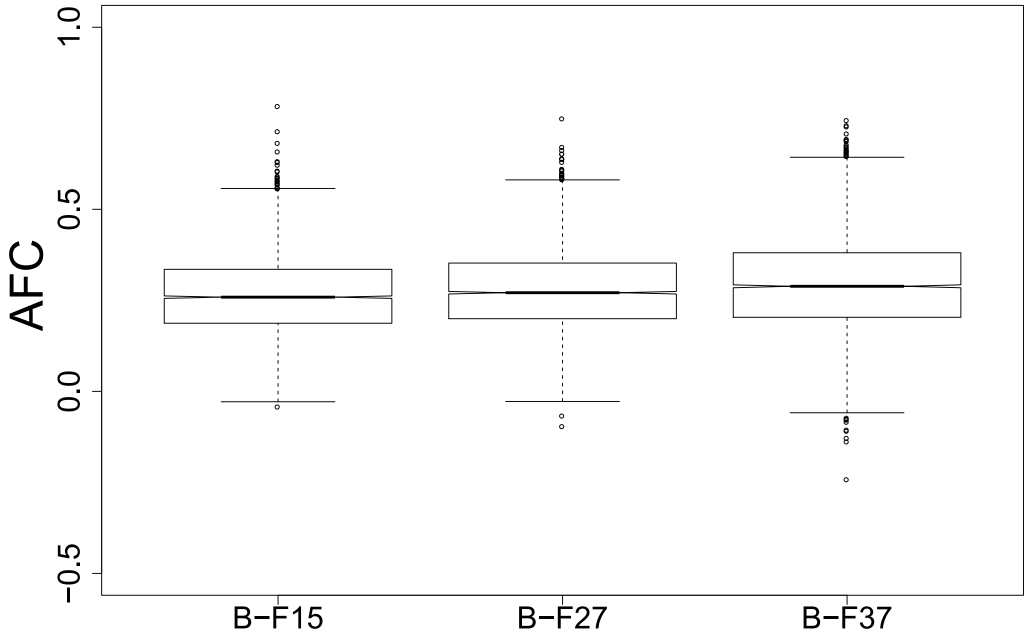

B)

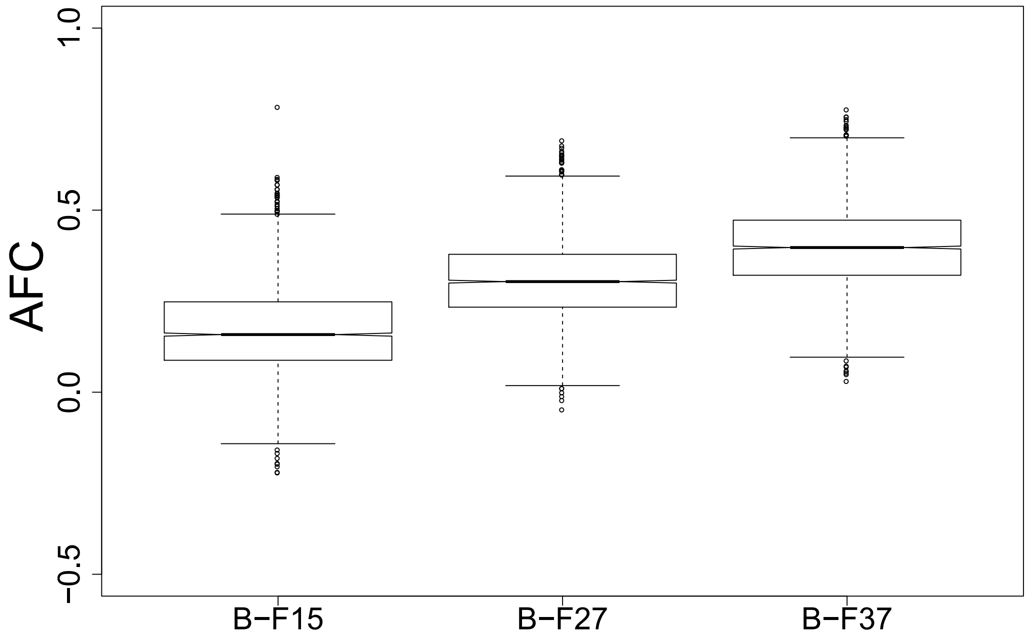

Fig. 4

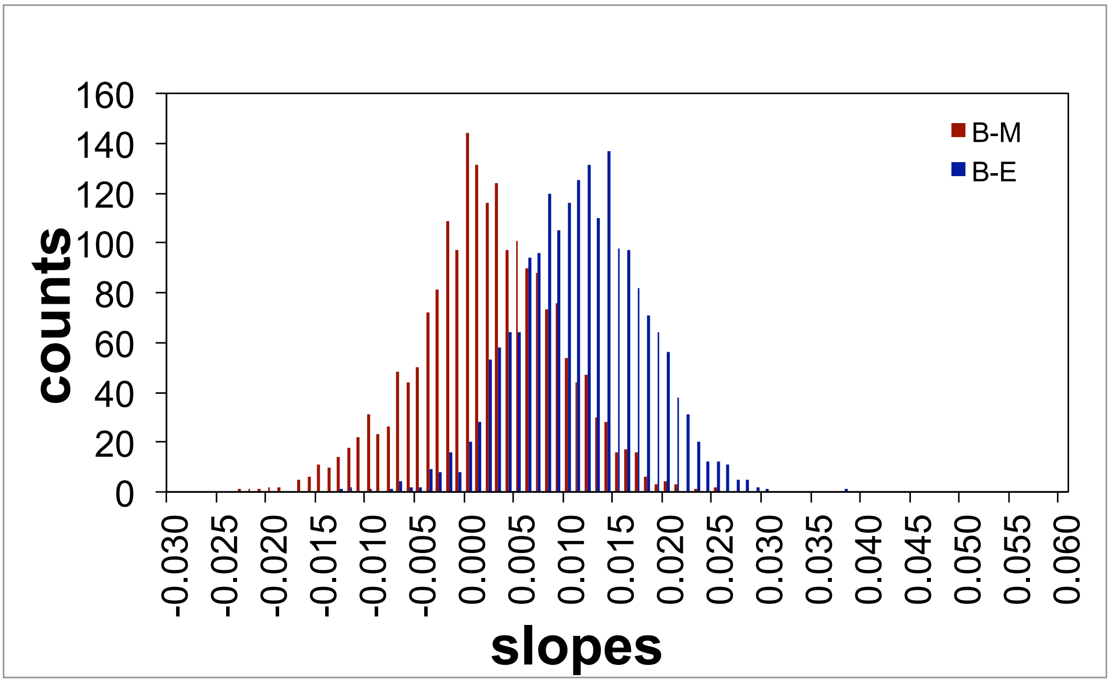
B-M
B-E
counts
160
140
120
100
80
60
40
20
0
-0.030
-0.025
-0.020
-0.015
-0.010
-0.005
0.000
0.005
0.010
0.015
0.020
0.025
0.030
0.035
0.040
0.045
0.050
0.055
0.060
slopes

360 Fig. 5

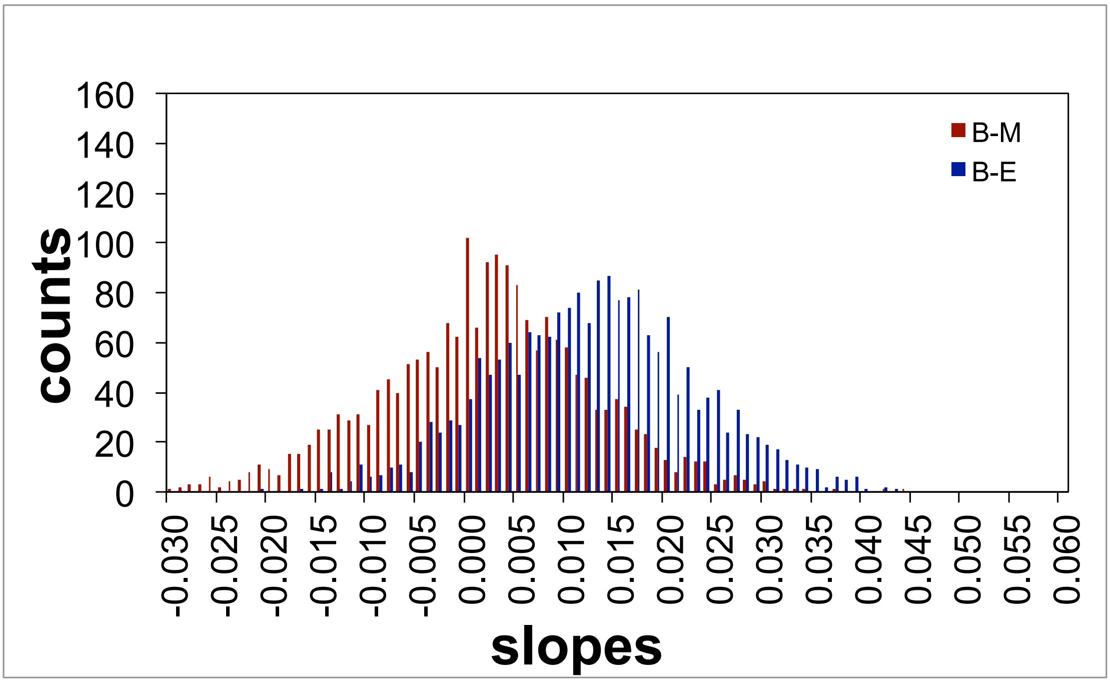

361

362

Fig. 6

A)

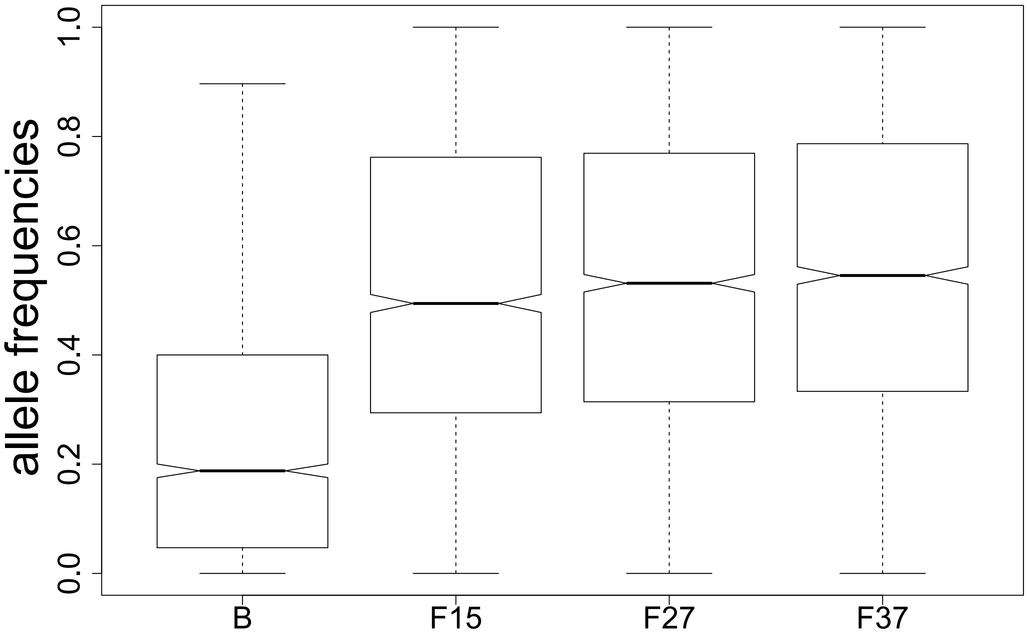
allele frequencies
0.0
0.2
0.4
0.6
0.8
1.0
B
F15
F27
F37

B

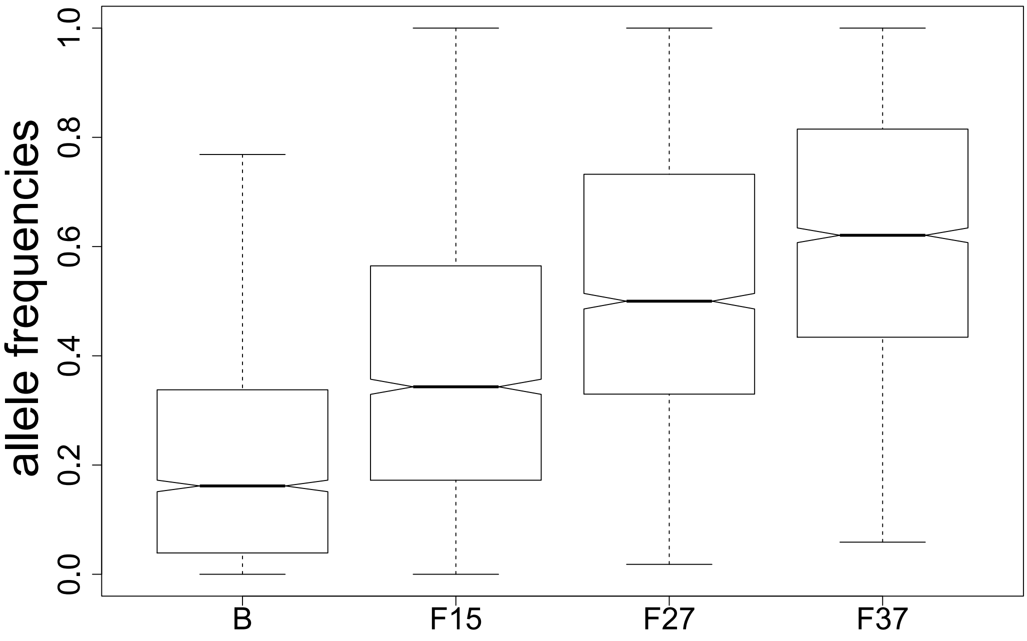
allele frequencies
0.0
0.2
0.4
0.6
0.8
1.0
B
F15
F27
F37

C)

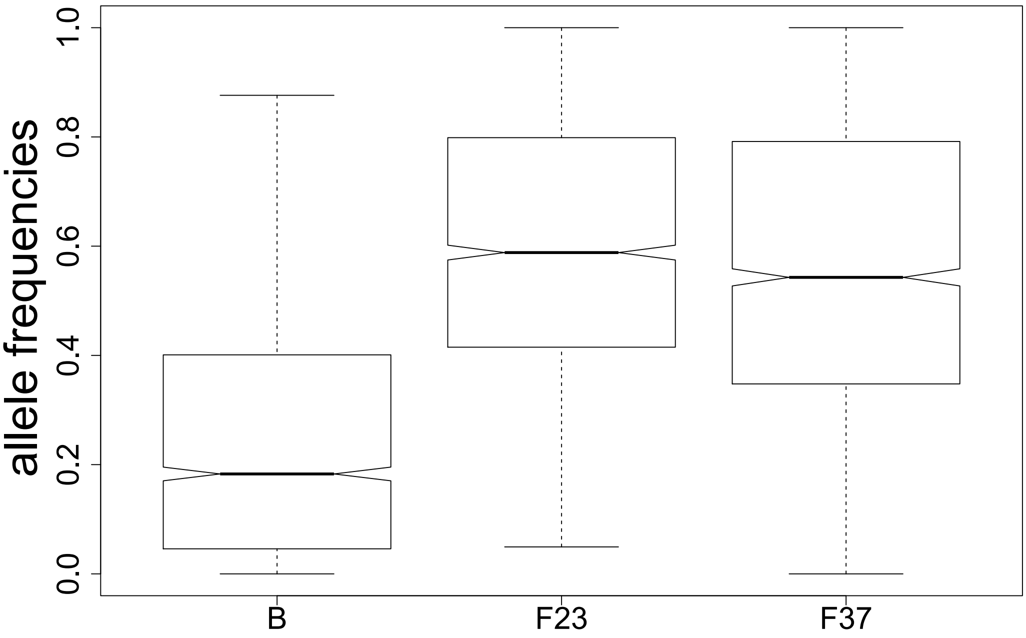
allele frequencies
0.0
0.2
0.4
0.6
0.8
1.0
B
F23
F37

D)

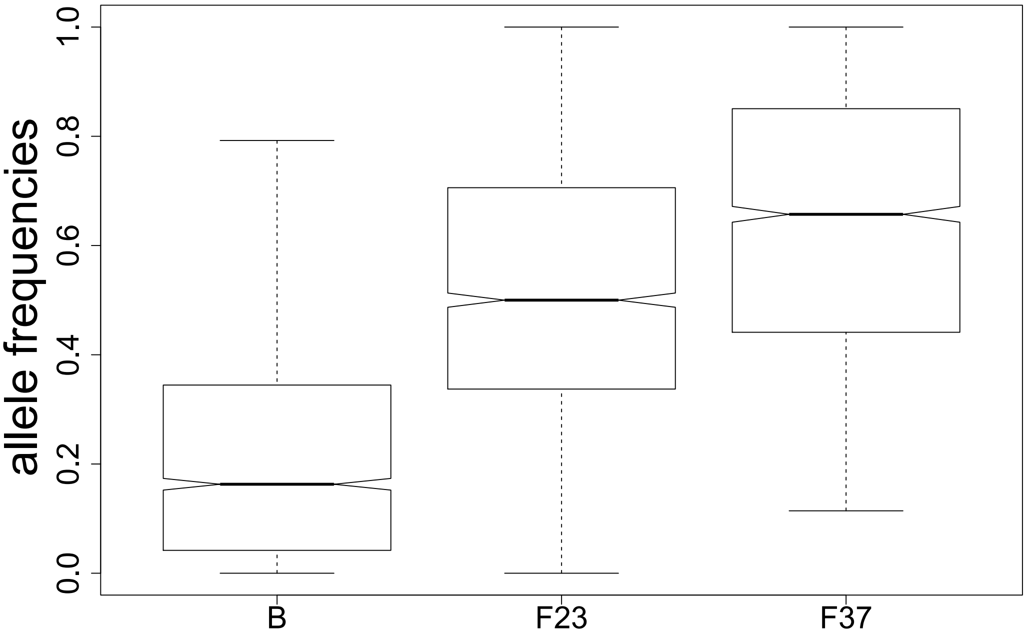
allele frequencies
0.0
0.2
0.4
0.6
0.8
1.0
B
F23
F37

E)

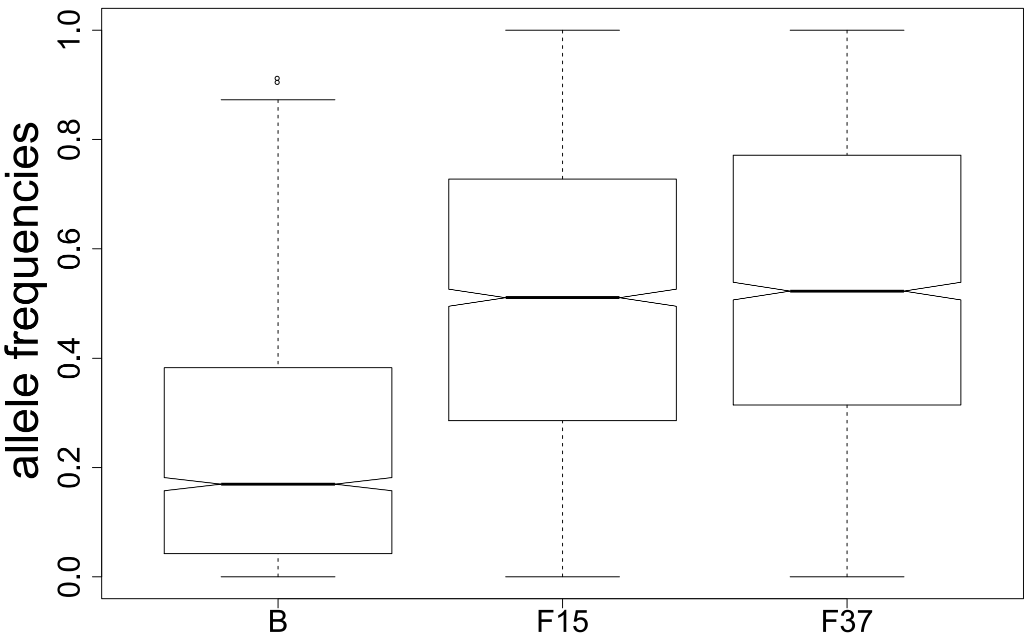
allele frequencies
0.0
0.2
0.4
0.6
0.8
1.0
B
F15
F37

F)

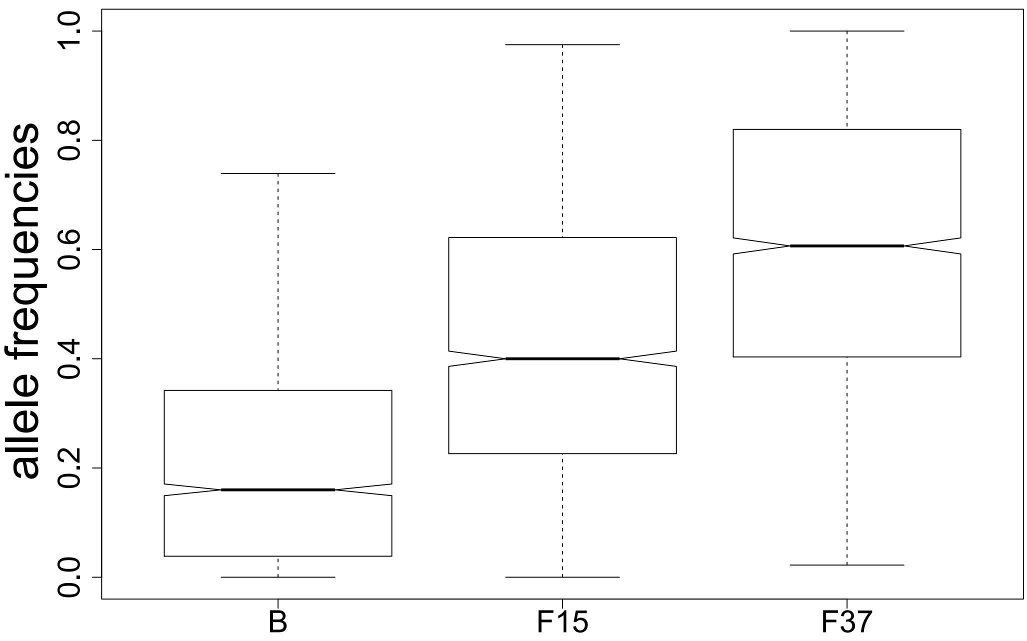
allele frequencies
0.0
0.2
0.4
0.6
0.8
1.0
B
F15
F37

Fig. 7

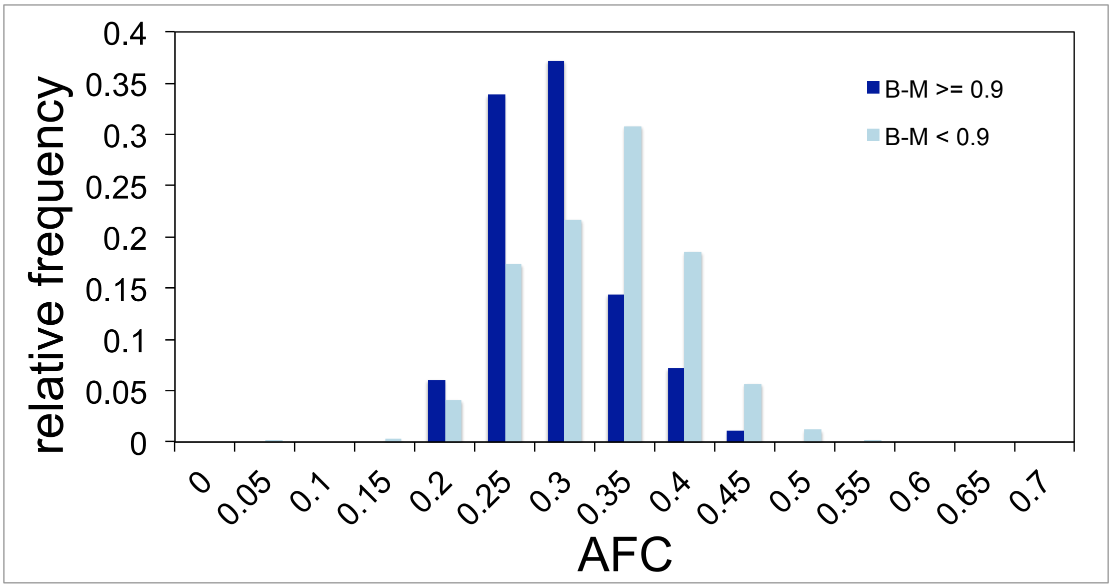
relative frequency
0.4
0.35
0.3
0.25
0.2
0.15
0.1
0.05
0
B-M >= 0.9
B-M < 0.9
0
0.05
0.1
0.15
0.2
0.25
0.3
0.35
0.4
0.45
0.5
0.55
0.6
0.65
0.7
AFC

Fig. 8

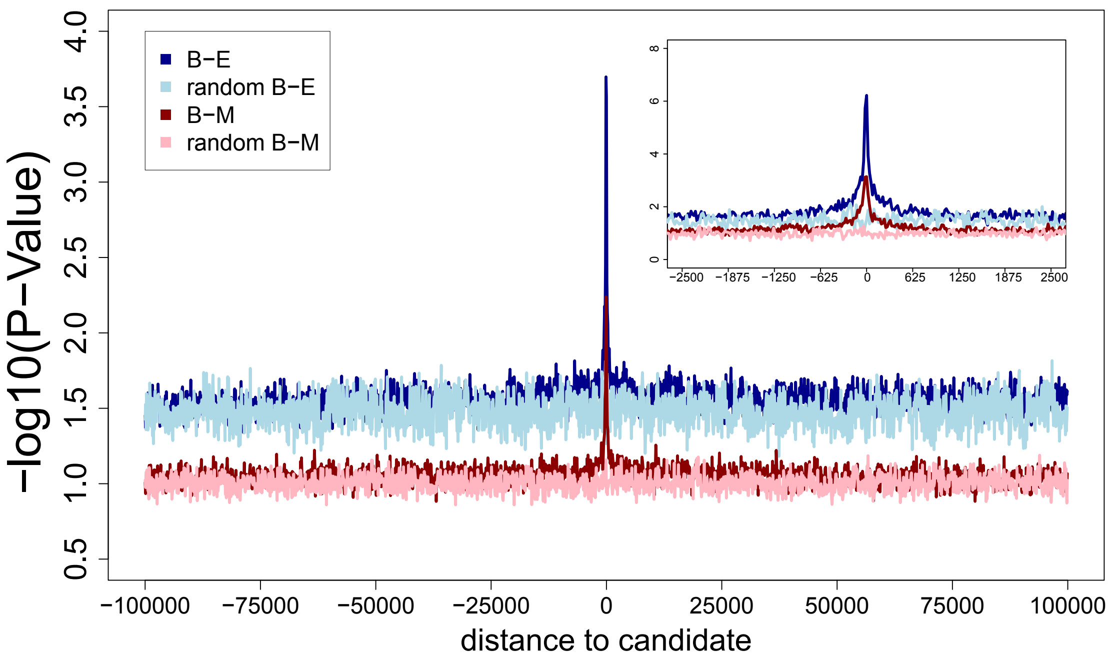
B−E
random B−E
B−M
random B−M
−log10(P−Value)
0.5
1.0
1.5
2.0
2.5
3.0
3.5
4.0
−100000
−75000
−50000
−25000
0
25000
50000
75000
100000
distance to candidate
0
2
4
6
8
−2500
−1875
−1250
−625
0
625
1250
1875
2500

Fig. 9

A)

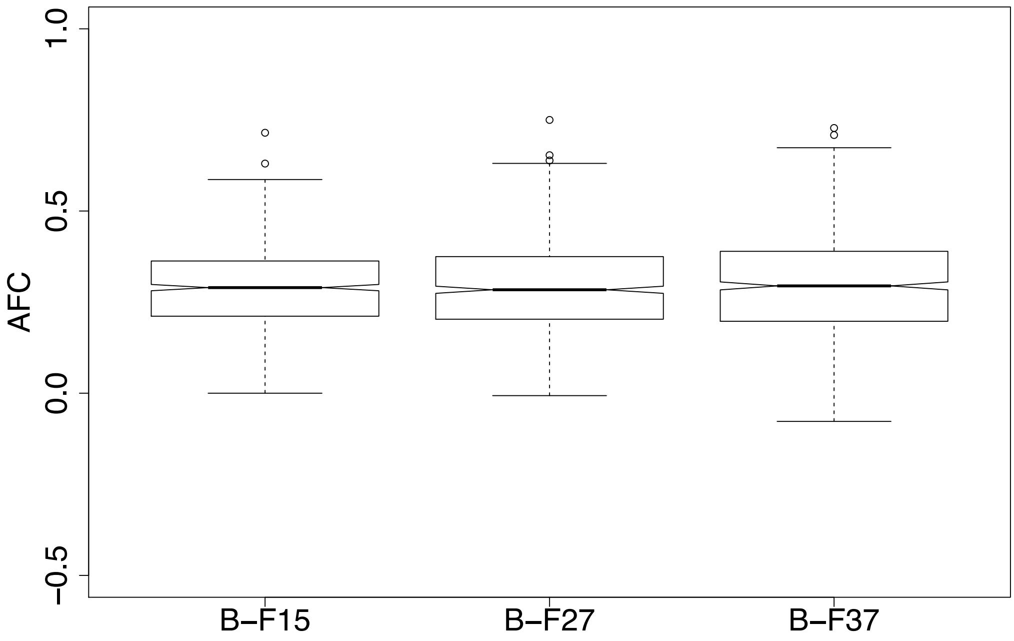

B)

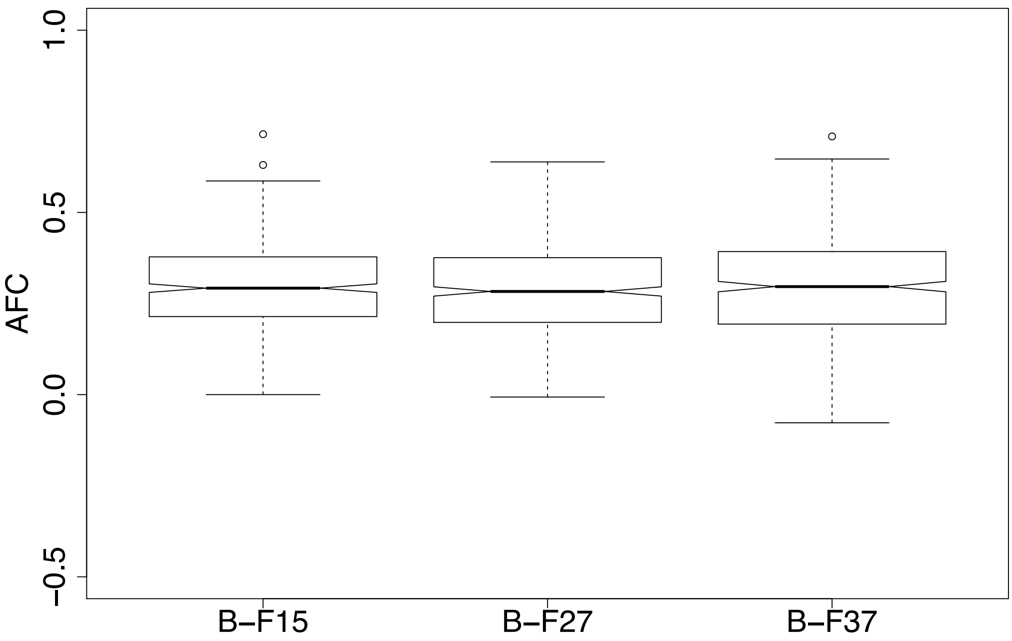

Fig. 10

A)

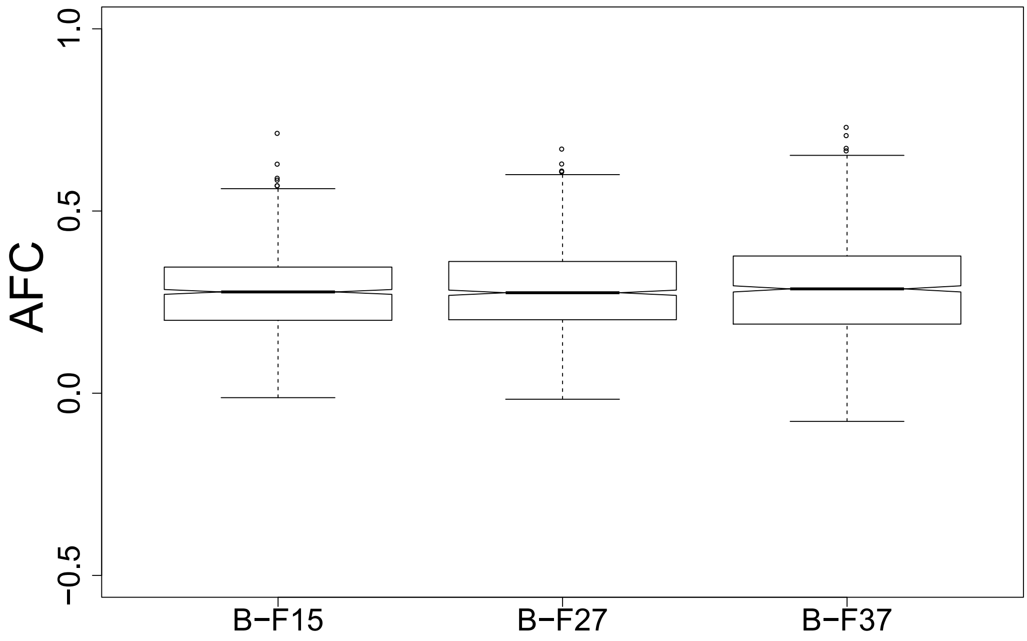

B)

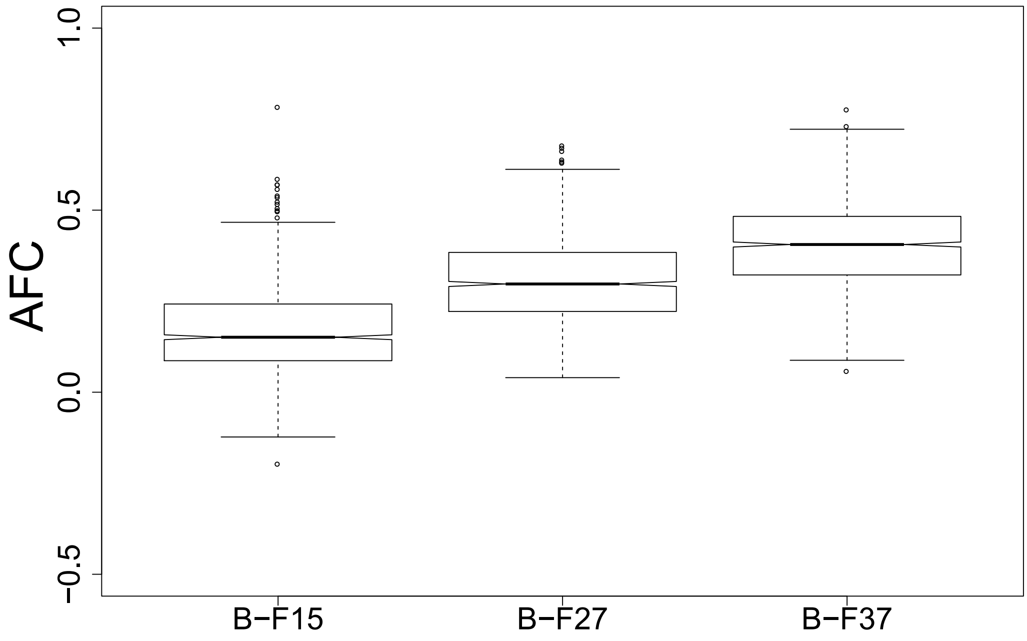

Fig. 11

A

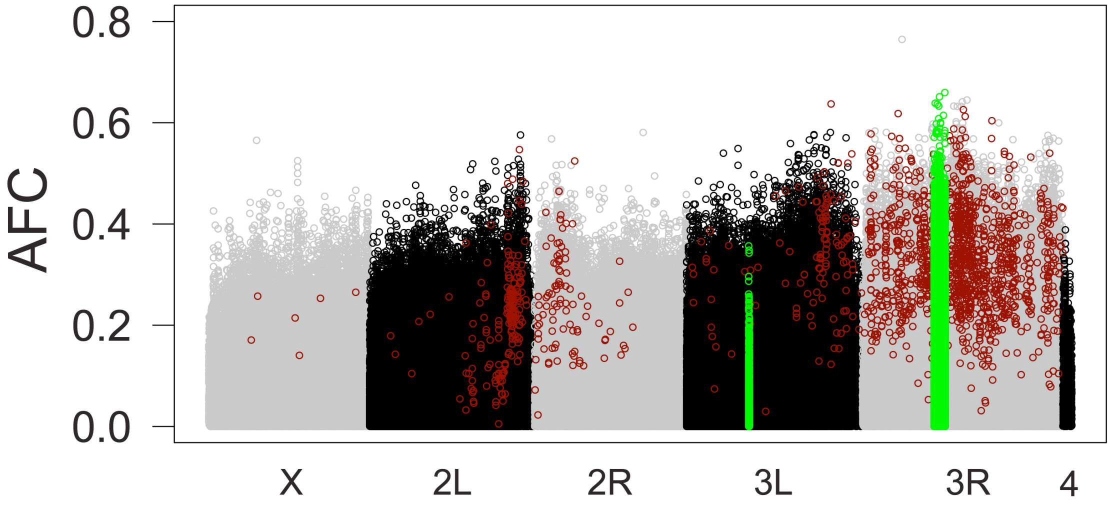

B

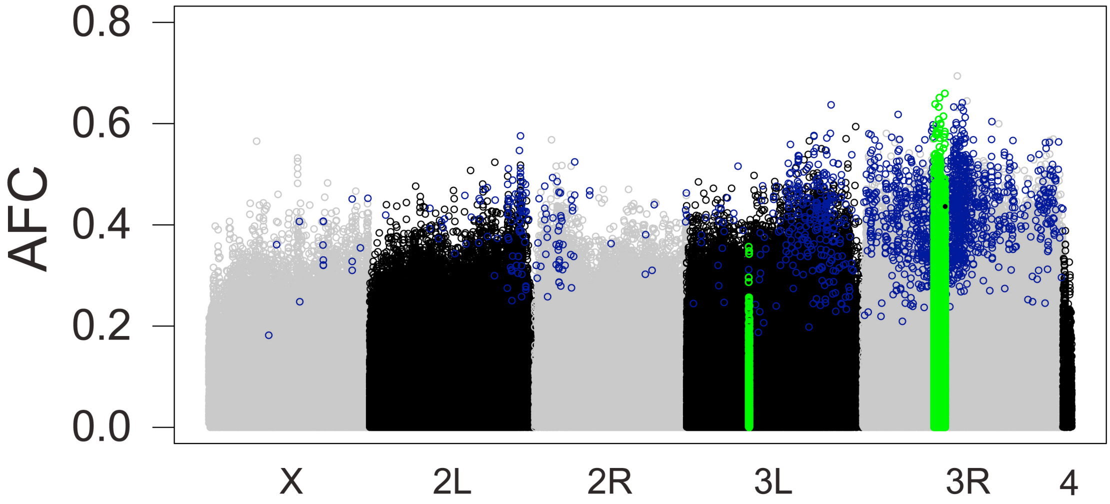

C

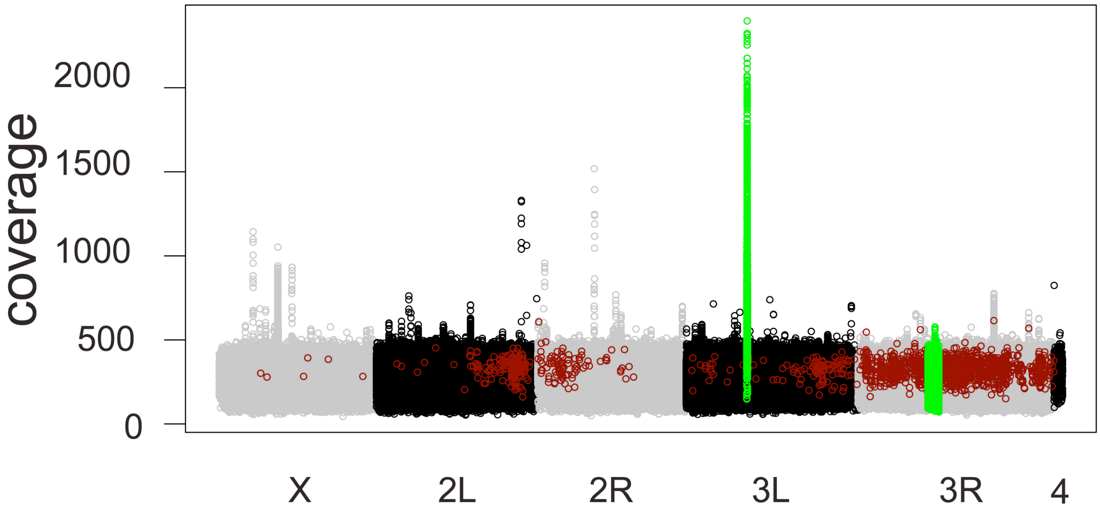
coverage
2000
1500
1000
500
0
X
2L
2R
3L
3R
4

D

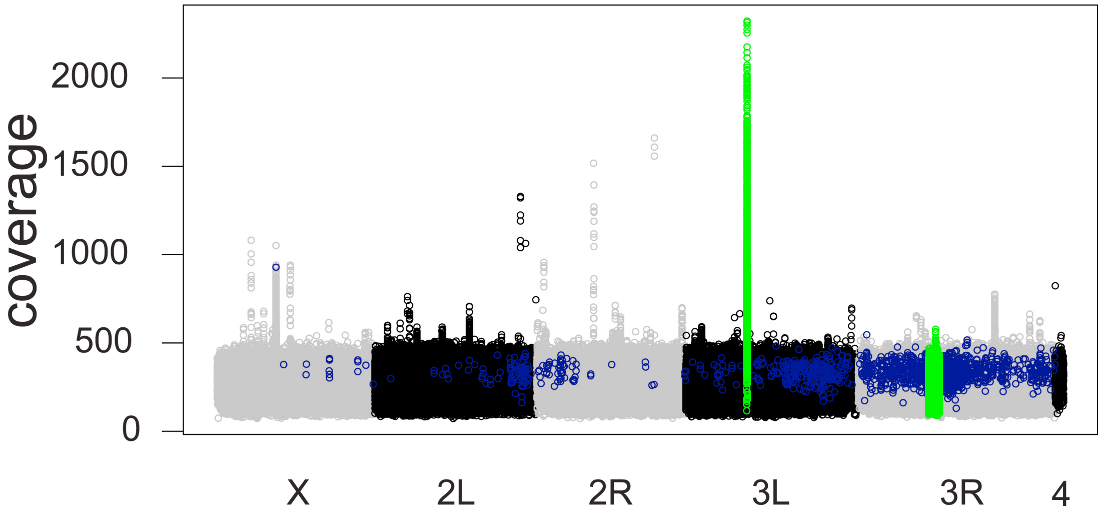
coverage
2000
1500
1000
500
0
X
2L
2R
3L
3R
4

Fig. 12

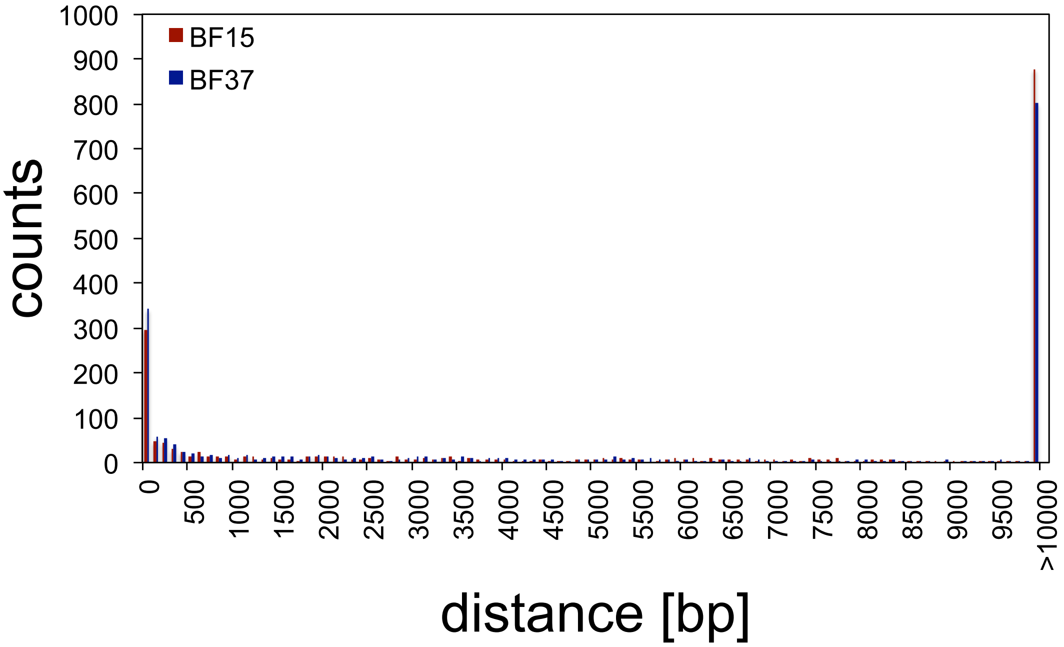
BF15
BF37
counts
1000
900
800
700
600
500
400
300
200
100
0
0
500
1000
1500
2000
2500
3000
3500
4000
4500
5000
5500
6000
6500
7000
7500
8000
8500
9000
9500
>10000
distance [bp]

Fig. 13

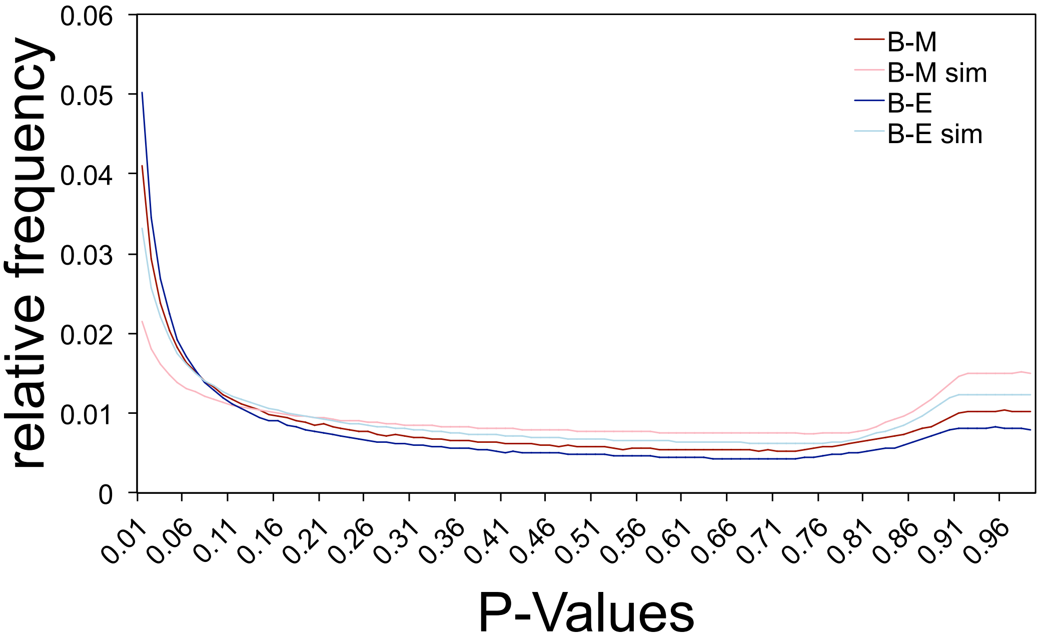

relative frequency
0.06
0.05
0.04
0.03
0.02
0.01
0
B-M
B-M sim
B-E
B-E sim
0.01
0.06
0.11
0.16
0.21
0.26
0.31
0.36
0.41
0.46
0.51
0.56
0.61
0.66
0.71
0.76
0.81
0.86
0.91
0.96
P-Values

Fig. 14

A

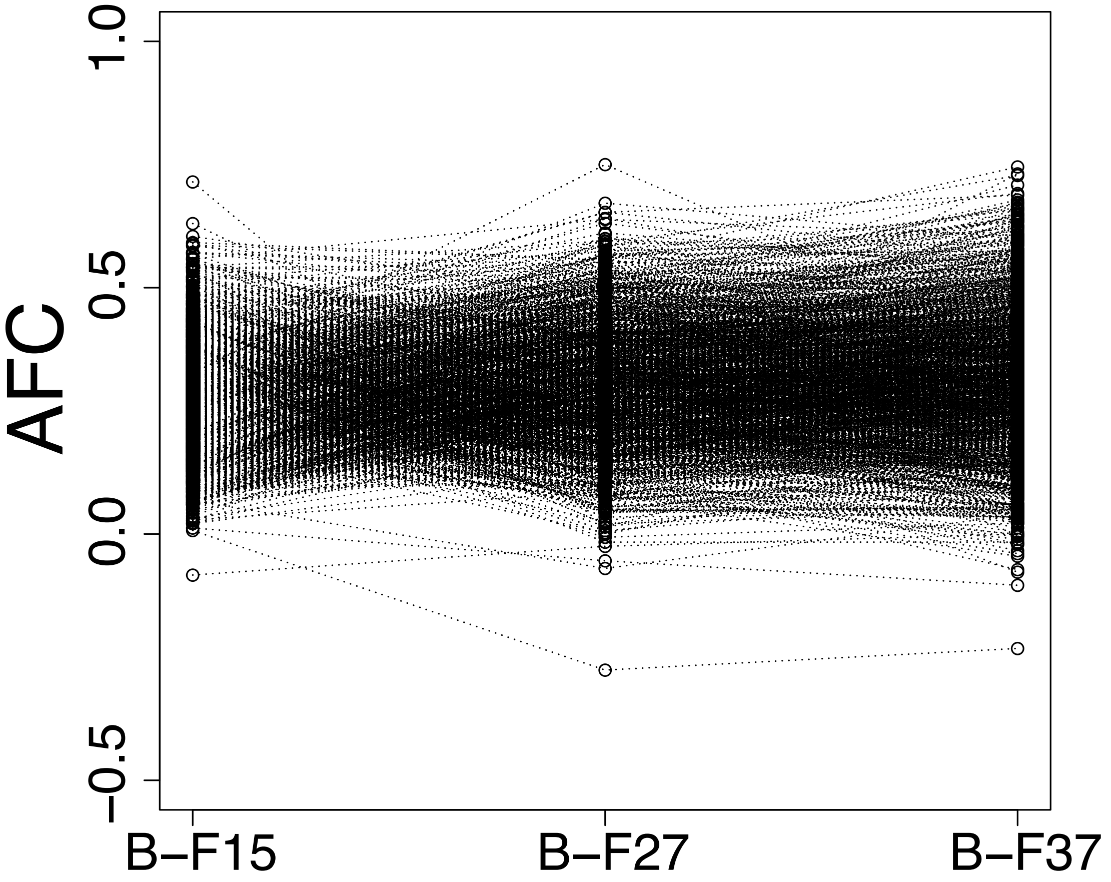

B

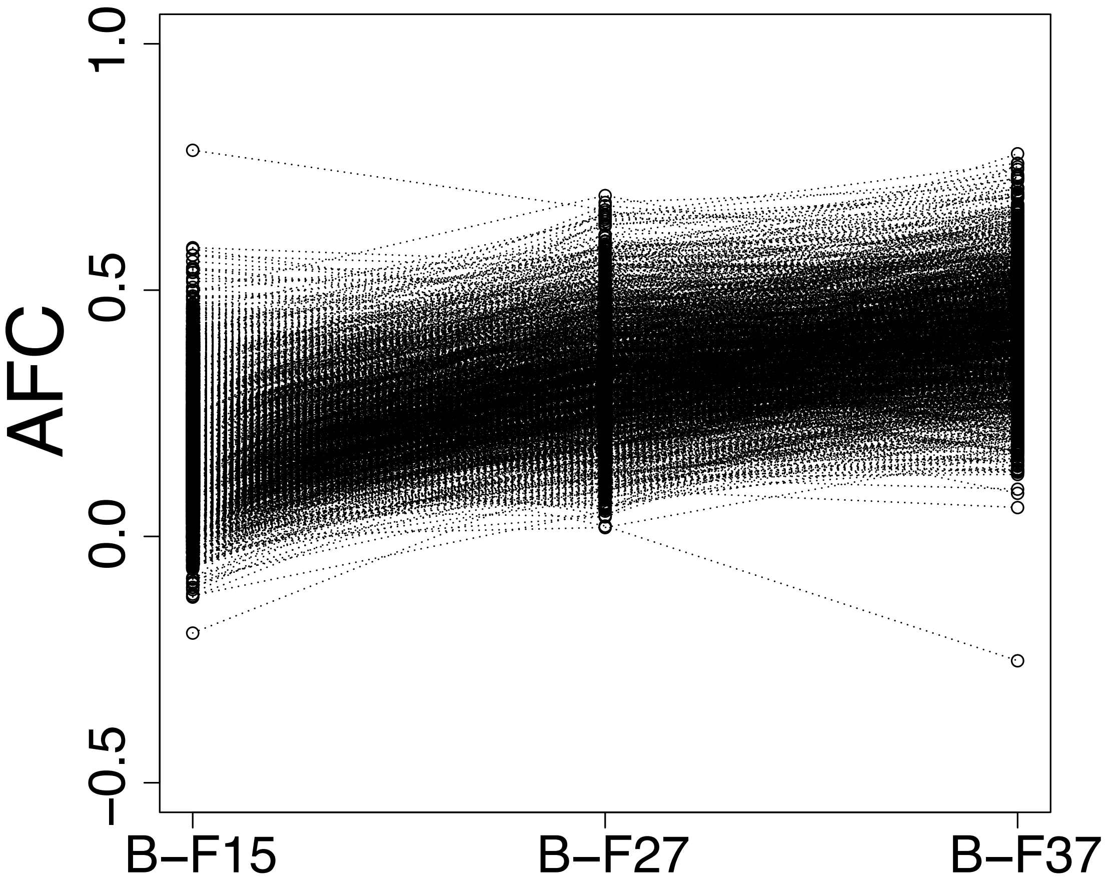

Supp. Table 1: Coverage for each population

generation	replicate	mean coverage
0	1	64
0	2	30
0	3	55
15	2	50
15	3	45
23	1	41
27	3	55
37	1	30
37	2	30
37	3	30

Supp. Table 2: π estimates for each chromosome arm and population (replicate 3)

	**Base population**	**F 37**
X	0.00345	0.00341
2L	0.00708	0.00686
2R	0.00550	0.00510
3L	0.00594	0.00511
3R	0.00538	0.00458
4	0.00075	0.00072

Supp. Table 3: Distribution of all SNPs and the 2000 most significant SNPS for the comparison B-M and B-E

	B-M				***B-E***			
	All SNPs		***Significant SNPs***		***All SNPs***		***Significant SNPs***	
**Chromosome**	**count**	**fraction**	**count**	**fraction**	**count**	**fraction**	**count**	**fraction**
X	181646	11.16%	6	0.30%	170827	11.25%	18	0.90%
2L	410267	25.20%	232	11.60%	386065	25.42%	102	5.10%
2R	287394	17.66%	121	6.05%	267927	17.64%	72	3.60%
3L	369552	22.70%	146	7.30%	341889	22.51%	347	17.35%
3R	373089	22.92%	1491	74.55%	346593	22.82%	1460	73.00%
4	1991	0.12%	0	0.00%	1945	0.13%	0	0.00%
other	3840	0.002359043	4	0.20%	3462	0.23%	1	0.05%
**total**	**1627779**	**100.00%**	**2000**	**100.00%**	**1518708**	**100%**	**2000**	**100%**

Supp. Table 4: Distribution of all SNPs and the 2000 most significant SNPS on chromosome 3R for the comparison B-M and B-E

	B-M				***B-E***			
	All SNPs		***Significant SNPs***		***All SNPs***		***Significant SNPs***	
**location**	**count**	**fraction**	**count**	**fraction**	**count**	**fraction**	**count**	**fraction**
Inside *In(3R)Payne*	149829	40.16%	783	52.52%	141333	40.78%	666	45.62%
Outside *In(3R)Payne*	223260	59.84%	708	47.48%	205260	59.22%	794	54.38%
**3R**	**373089**	**100.00%**	**1491**	**100.00%**	**346593**	**100.00%**	**1460**	**100.00%**

Supp. Table 5: Frequency of the inversion *In(3R)Payne* determined by PCR

Population	***Frequency***
Base population replicate 1	0.11
F18 replicate 1	0.04
F38 replicate 1	0.02
F 38 replicate 2	0.01

Supp. Table 6: Distribution of all SNPs and the 2000 most significant SNPs split by genomic feature for the comparison B-M and B-E. All categories were tested for significant over- or underrepresentation of candidate SNPs by X^2 tests with one degree of freedom. *P*-Values thresholds were Bonferroni-corrected for multiple testing (α = 0.0056). Bold categories are significantly over- (indicated by $^{-}$) or underrepresented (indicated by $^{+}$).

	B-M				***B-E***			
	full		***candidate***		***full***		***candidate***	
**SNP_Effect**	**count**	**fraction**	**count**	**fraction**	**count**	**fraction**	**count**	**fraction**
3PRIME_UTR	58996	3.32%	71	3.24%	**55463**	**3.35%**	**102**	**4.68%$^{+}$**
5PRIME_UTR	37721	2.13%	51	2.33%	34696	2.10%	49	2.25%
DOWNSTREAM	52924	2.98%	61	2.78%	**50381**	**3.04%**	**93**	**4.26%$^{+}$**
INTERGENIC	**510045**	**28.74%**	**532**	**24.27%$^{-}$**	477806	28.86%	623	28.56%
INTRON	**801289**	**45.15%**	**1102**	**50.27%$^{+}$**	**750478**	**45.33%**	**1065**	**48.83%$^{+}$**
NON_SYNONYMOUS_CODING	**65541**	**3.69%**	**116**	**5.29%$^{+}$**	59015	3.56%	87	3.99%
SYNONYMOUS_CODING	**179425**	**10.11%**	**166**	**7.57%$^{-}$**	**162898**	**9.84%**	**86**	**3.94%$^{-}$**
UPSTREAM	61438	3.46%	88	4.01%	58077	3.51%	67	3.07%
other	7434	0.42%	5	0.23%	6800	0.41%	9	0.41%
**total**	**1774813**	**100.00%**	**2192**	**100.00%**	**1655614**	**100.00%**	**2181**	**100.00%**

Supp. Table 7: Median allele frequency changes of all pairwise population comparisons in replicate one of the top 2000 candidates from the cage experiment and the top 2000 candidates of the simulated data.

	**B-F15**	**B-F27**	**B-F37**
B-M	0.282	0.296	0.309
B-M sim	0.206	0.087	0.128
B-E	0.167	0.319	0.417
B-E sim	0.085	0.126	0.304

Supp. Table 8: Table of overrepresented GO Terms with an FDR ≤ 0.4 for datasets B-M and B-E. Column 1 shows the name of the GO category; Column 2 the expected number of genes found in this category from simulations; and Column 3 the observed number of genes from the data. Column 4 and 5 show the *P*-value and FDR, respectively. The last two columns show a description of the GO category and the names of the candidate genes detected for this category. See GO.xls
