# Supplementary figures and images for: Adaptation of Drosophila to a novel laboratory environment reveals temporally heterogeneous trajectories of selected alleles
Source: Mol Ecol. 2012 Oct;21(20):4931–41. doi: 10.1111/j.1365-294X.2012.05673.x (PMC3533796; doi:10.1111/j.1365-294X.2012.05673.x)

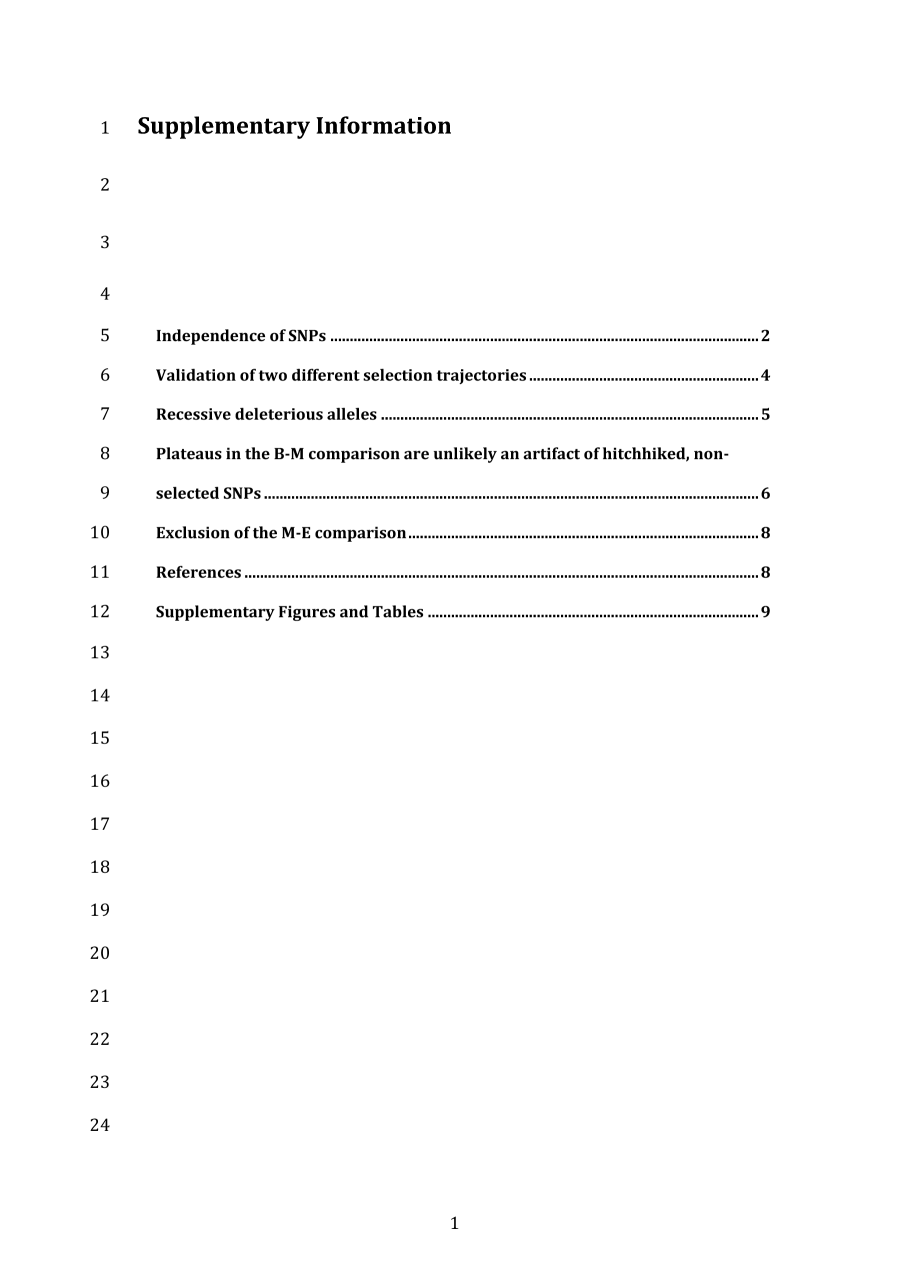

Supplement: Supplementary file 3 [file mec0021-4931-SD3.png]
